# Quantum-like Qualia hypothesis: from quantum cognition to quantum perception

**DOI:** 10.3389/fpsyg.2024.1406459

**Published:** 2025-04-03

**Authors:** Naotsugu Tsuchiya, Peter Bruza, Makiko Yamada, Hayato Saigo, Emmanuel M. Pothos

**Affiliations:** ^1^Faculty of Medicine, Nursing, and Health Sciences, School of Psychological Sciences, Turner Institute for Brain and Mental Health, Monash University, Melbourne, VIC, Australia; ^2^Center for Information and Neural Networks (CiNet), National Institute of Information and Communications Technology (NICT), Suita-shi, Osaka, Japan; ^3^Laboratory of Qualia Structure, ATR Computational Neuroscience Laboratories, Kyoto, Japan; ^4^School of Information Systems, Queensland University of Technology, Brisbane, QLD, Australia; ^5^National Institutes for Quantum and Radiological Science and Technology, Chiba, Japan; ^6^Nagahama Institute of Bio-Science and Technology, Nagahama, Japan; ^7^Department of Psychology, City, University of London, London, United Kingdom

**Keywords:** qualia, quantum cognition, consciousness, attention, similarity, Bell inequality, bistable perception

## Abstract

To arbitrate theories of consciousness, scientists need to understand mathematical structures of quality of consciousness, or qualia. The dominant view regards qualia as points in a dimensional space. This view implicitly assumes that qualia can be measured without any effect on them. This contrasts with intuitions and empirical findings to show that by means of internal attention qualia can change when they are measured. What is a proper mathematical structure for entities that are affected by the act of measurement? Here we propose the mathematical structure used in quantum theory, in which we consider qualia as “observables” (i.e., entities that can, in principle, be observed), sensory inputs and internal attention as “states” that specify the context that a measurement takes place, and “measurement outcomes” with probabilities that qualia observables take particular values. Based on this mathematical structure, the Quantum-like Qualia (QQ) hypothesis proposes that qualia observables interact with the world, as if through an interface of sensory inputs and internal attention. We argue that this qualia-interface-world scheme has the same mathematical structure as observables-states-environment in quantum theory. Moreover, within this structure, the concept of a “measurement instrument” in quantum theory can precisely model how measurements affect qualia observables and states. We argue that QQ naturally explains known properties of qualia and predicts that qualia are sometimes indeterminate. Such predictions can be empirically determined by the presence of order effects or violations of Bell inequalities. Confirmation of such predictions substantiates our overarching claim that the mathematical structure of QQ will offer novel insights into the nature of consciousness.

## Highlights

- The recent explosion in theories of consciousness, which aim to link subjectivity and physical substrates, require a better characterization of mathematical structure of quality of consciousness, or qualia.
- In traditional and intuitive models of qualia, a particular quale is assumed to be a point in a high dimensional space.
- Such models assume that qualia exist independent of measurements, but they are incompatible with the findings that qualia are generally affected by measurements.
- To account for how the measurement can affect qualia, a Quantum-like Qualia (QQ) hypothesis proposes a mathematical structure employed in quantum theory.
- We will outline how QQ can be tested with various experimental paradigms, building on the successful quantum cognition framework.

## 1 Introduction

Research on consciousness has recently entered a new phase. A burst of neuroimaging studies on consciousness since 1990 has produced a huge amount of empirical data, requiring a principled explanation for consciousness and its neuronal substrate (Koch et al., 2016; Mashour et al., 2020; Seth and Bayne, 2022). Over the last 20 years, many of the initial ideas about consciousness and brains were abandoned in the face of empirical data. The remaining theories have retained their core principles in the form of variations that have branched out from these theories. Some theories aspire to make quantitative predictions, a few of which are currently pitted against each other in an adversarial way (Melloni et al., 2021). Through empirical tests of rival theoretical predictions, substantial scientific progress is to be expected, as has happened in other fields, such as physics and experimental psychology (Einstein et al., 1935; Bell, 1964; Freedman and Clauser, 1972; Aspect et al., 1982; Kahneman, 2003).

As the science of consciousness matures, it has become increasingly clear that we lack an understanding of the target phenomenon, namely consciousness. While “consciousness” can mean the level or presence of consciousness, as in the clinical science of coma, general anesthesia, or deep sleep (Casarotto et al., 2016), this article focuses on the issue of quality of consciousness, feelings of what-it-is-like-to-be, or, in short, qualia (Balduzzi and Tononi, 2009; Kanai and Tsuchiya, 2012; Tsuchiya and Saigo, 2021; Tye, 2021; Lyre, 2022). Qualia in consciousness research comes in two senses, broad and narrow. In the broad sense, we use a quale to mean a moment of entire conscious experience across all sensory modalities and thoughts, that is, everything being experienced. Qualia in the narrow sense refers to one aspect of the experience, such as the “redness” of the sunset, the particular flavor and taste of tuna sashimi, and so on (Balduzzi and Tononi, 2009; Kanai and Tsuchiya, 2012). This article embraces both senses of qualia. What is not qualia concerns everything that is not part of our conscious experience.

In this article, Section 2 reviews the popular models of qualia and their deficiencies. To address these deficiencies, Section 3 proposes the Quantum-like Qualia (QQ) hypothesis. Our hypothesis is inspired by the mathematical structure of quantum theory. None of our claims rests on whether or not microscopic quantum phenomena play a significant role in the brain and/or consciousness. Section 4 focuses on empirical research projects that can test the validity of the QQ hypothesis, followed by the conclusion in Section 5.

## 2 Traditional qualia models and their deficiencies

**Figure 1:**
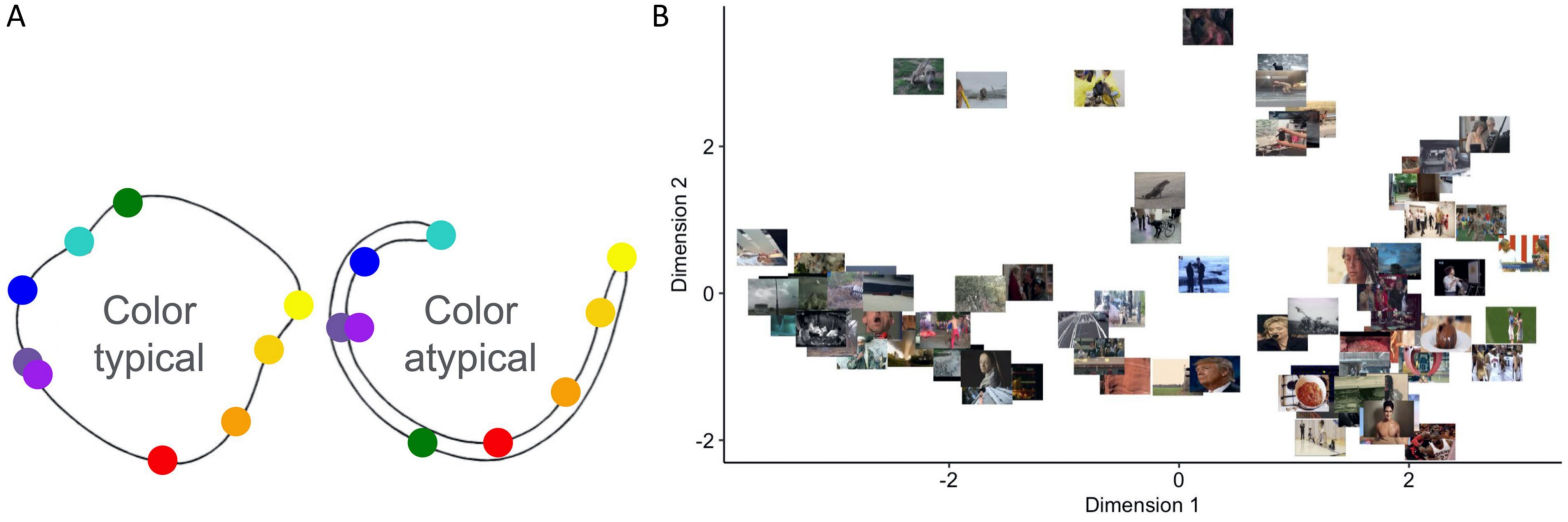
Traditional psychological space models. Traditional psychological space models (Shepard and Cooper, 1992; Rosenthal, 2015; Lee, 2021) assume each quale occupies a point in space (or a combination of points). “Distances” between two points are assumed to be related to perceived experiential similarity (Krumhansl, 1978; Ashby and Perrin, 1988; Nosofsky, 1991). **(A)** A classic color hue ring model for the representation of similarity relationships among 9 colors for color-typical and red-green color blind individuals. Modified from Shepard and Cooper (1992). **(B)** Similar representations (points-in-high dimensional spaces) have been used in other domains of experience, such as emotional experience. Adapted from Lin (2023), which used the emotional movie stimuli in Cowen and Keltner (2017).

Traditional models of qualia are founded on the notion of points in a putative metric space, sometimes called a psychological space, quality space, qualia space, phenomenal space (Clark, 2000; Rosenthal, 2015; Lee, 2021; Figure 1A). These models have been proposed for various modalities, such as color, time, pain, sound and smell (Shepard and Cooper, 1992; Churchland, 2005; Klincewicz, 2011; Kostic, 2012; Young et al., 2014; Renero, 2014). In the cognitive domain, there are strong arguments that concepts reside in such a space (Gärdenfors, 2000). Thus it seems natural to start with the idea to represent qualia as single points in a high dimensional space. Here, a definite point corresponds to a particular quale (either in the narrow or broad sense). To specify a combination of narrow qualia or a quale in the broad sense, multiple points are often considered as well.^1^

In the case of narrow sense qualia, the distance between the two points relates to the “similarity” between the respective qualia (e.g., a red quale and an orange quale are close in similarity, but red and green are dissimilar). Inspired by early work by Shepard, many variants of such similarity models have been proposed (Krumhansl, 1978; Ashby and Perrin, 1988; Nosofsky, 1991), where visualization techniques such as multidimensional scaling (Borg and Groenen, 2005) have played a central role (Figures 1A,B). Under this framework, various types of qualia, e.g., color (Indow, 1988; Shepard and Cooper, 1992; Churchland, 2005; Bujack et al., 2022; Zeleznikow-Johnston et al., 2023), sound (Shepard, 1982; Renero, 2014; Cowen et al., 2020), object (Hebart et al., 2020), emotion (Figure 1B) (Cowen and Keltner, 2017; Nummenmaa et al., 2018), olfaction (Young et al., 2014), art (Graham et al., 2010) etc., have been investigated and visualized based on similarity ratings of pairwise comparisons between the set of qualia under investigation.

Despite widespread use, the psychological space approach to modeling qualia encounters three challenges: the inability to adequately capture indeterminate and dynamic facets of qualia, as well as their intricate interactions with internal mental processes. The following summary briefly covers these three points.

Firstly, as this approach assumes a quale is a definite entity (e.g., a point or points in a space), it is unable to capture the intuition that some qualia appear to be indeterminate entities. The indeterminacy of qualia becomes apparent when one introspects on the border of experience in space or time or the nature of unattended or barely attended experience. To determine the spatial border of experience, one can stretch their arms to estimate the limit of the visual field at the periphery, and experientially confirm that this limit is tenuous. Under complete darkness, it is not clear that any such boundary exists. Time also seems to have an indeterminate character. The start and end times of an event often feel unsure and a moment rarely feels point-like, but is typically experienced as having some duration (Filk, 2013). Even when one is focally attending to qualia, one can sense an uncertainty regarding the phenomenal appearance. Changes in certain aspects of qualia have been psychophysically confirmed. The very act of attending can alter the quality of the experience (Carrasco and Barbot, 2019).

Qualia can be uncertain in two ways. Firstly, the “epistemic” uncertainty of qualia implies that qualia themselves are always determinate, i.e., in a definite state, but measurement processes inject noise so that there is uncertainty about the value of this definite state. Epistemic uncertainty can be captured by modifying the classical model by replacing a point with a cloud of points. However, we suspect that some qualia are “ontologically” indeterminate. Such qualia can be characterized as being in an indefinite “state” whereby properties can only be attributed by means of measuring an ensemble of like qualia. Consequently, indeterminate qualia cannot be modeled or represented as a cloud of dots.

Secondly, the psychological space approach is by default static and does not account for the temporal dynamics of qualia, because it maps sensory inputs into qualia “at a given time” (see also Footnote 1). The temporal dynamics of qualia, however, are one of the most studied aspects of qualia, from very fine time scales using masking and priming (Bachmann, 2000; Breitmeyer and Ogmen, 2007), to larger time scales involving adaptation, expectation (Melloni et al., 2011), and multistability (Maier et al., 2012; Brascamp et al., 2018). If the space itself changes dynamically, the traditional psychological space approach may require substantial updates to account for the spatio-temporal dynamics of qualia.

Thirdly, the psychological space approach is not well developed regarding how qualia interact with internal mental processes, such as attention. As alluded to above, how we attend to sensory inputs appears to significantly alter what we experience (Carrasco and Barbot, 2019), as implied from change blindness and inattentional blindness demonstrations (Simons and Rensink, 2005; Pitts et al., 2018). However, before we pay attention, we already experience something at the to-be-attended locations, and that is the reason why we can consciously direct attention there. The psychological space model is similarly unclear about how qualia relate to other internal processes, such as memory and expectation.

Of course, any general framework can be in principle extended. Yet, since the pioneering work by Shepard (1962a,b, 1970, 1980, 1987), subsequent extensions (e.g., concerning dynamics) have not been proposed. It is noteworthy that masking effects have been documented for over a century (Exner, 1868; Breitmeyer and Ogmen, 2007), and despite more than six decades of exploration within high dimensional point models, scant insights into these effects have emerged. We contend that the outlined QQ hypothesis presented here holds promise for explicating such masking phenomena, even without properly fleshed out computational models.^2^

Thus, the psychological space approach to modeling qualia as points in a dimensional space appears deficient in regard to psychophysically-informed intuitions that qualia are indeterminate, dynamic, and interact with other mental processes. But why do researchers continue to adhere to the psychological-space models? We surmise that this is due to the combination of the intuitive appeal of such models and the lack of compelling alternatives.^3^

Interestingly, a similar situation arose in the field of cognitive science, in particular decision making. In decision making, models based on standard probability theory and logic have been persistently challenged by many (apparently) paradoxical findings in human decision making. Some of these paradoxes in decision making have had fairly natural explanations by means of quantum probability theory, which was introduced in psychology with the quantum cognition framework (Khrennikov, 2010; Busemeyer and Bruza, 2024; Haven and Khrennikov, 2013; Pothos and Busemeyer, 2022).^4^ Notably, analogous qualia-related concerns have been raised in the context of human decision-making. By incorporating the indeterminacy inherent in quantum theory and acknowledging the role of measurement in determining the state within cognitive processes, it has become possible to more effectively model these phenomena, propelling the growth of the quantum cognition field. Consequently, we posit that quantum cognition establishes the conceptual and theoretical foundation of the Quantum-like Qualia hypothesis.

Decision making and other cognitive processes are inextricably linked to perception and sensation (Barsalou, 2010) and also appear to share basic neural processing architectures. Thus, it seems natural to consider the application of quantum probability theory as an alternate mathematical framework for qualia, in order to address the challenges for the psychological space approach.

## 3 The Quantum-like Qualia hypothesis

The three essential challenges for existing models for qualia (i.e., indeterminacy, dynamics, and interactions) are inherently related with the limitations in “classical” approaches. Classical approaches assume that qualia can be probed, observed, reported or “measured,” without affecting them. To consider a more general mathematical structure, it is useful to start with the assumption that such “measurements” necessarily affect qualia. How much these measurements affect qualia can vary depending on various factors.

**Figure 2:**
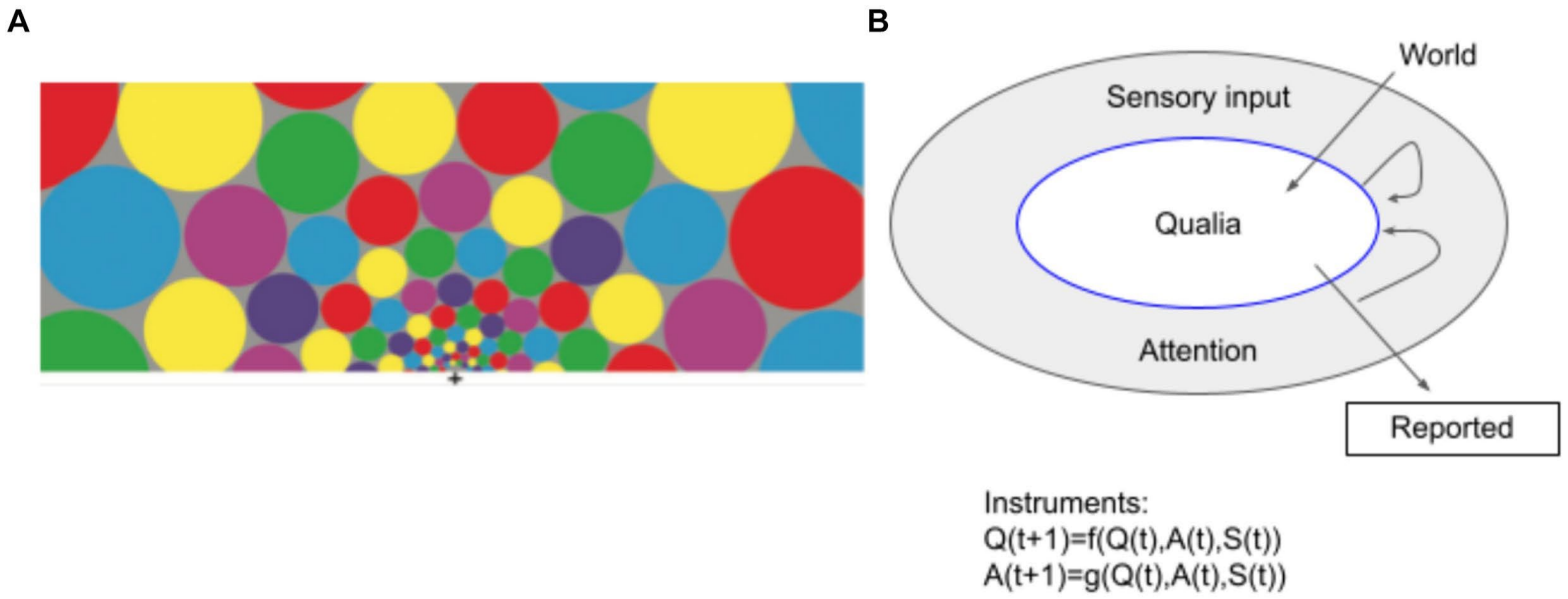
Conceptual framework of the QQ hypothesis. **(A)** An exemplar sensory input of many colorful patches with the size of each patch proportional to cortical magnification (Tyler, 2015). While you are fixating on the cross at the bottom center, you see the color of each patch without moving your eyes. However, you may feel your experience changes depending on where you direct your attention. **(B)** The conceptual diagram of QQ. QQ considers Qualia as observables that are properties of a system that can be in principle “measured,” probed and reported. Sensory inputs and Attention act as an interface or a “state” between Qualia and the World. For example, here the state can be “the sensory input as in **(A)** AND attending to a red patch on the right.” Then, we can define and measure a probability that a particular value is assigned to the observable, for example, Prob(“color Q for the leftmost circle” = “blue” | the state) =0.7. How Qualia (Q), Attention (A) and Sensory input (S) evolve over time with or without measurement is formalized by the theory of Instruments (Davies and Lewis, 1970; Ozawa and Khrennikov, 2021). Informally, the putative interaction between the world and qualia, qualia and subjective reports, and how reports alter attention and qualia through instruments are depicted by arrows in the panel.

Quantum theory offers a mathematical structure that deals with entities whose properties can change upon measurement. As we argue below, such a mathematical structure, proposed as a Quantum-like Qualia (QQ)^5^ hypothesis, attains the three desired features for qualia. QQ states that qualia are like quantum entities, which are inextricably affected by measurement. We first give a broad sketch of QQ (Figure 2), then explain technical concepts with familiar examples from consciousness research. More detailed mathematical formulations will be pursued in future work.

### 3.1 Separating qualia observables from states (of sensory input and attention)

To account for the indeterminacy of qualia, QQ distinguishes each instance of measured value of qualia (say, color qualia Q = “red”) from all possible measurable qualia. Inspired by quantum theory, we call all possible measured outcomes “observables.” Observables are intrinsic properties of a system that can, in principle, be measured. For example, a color qualia observable at the fixation can be a coarse set of color labels, such as Q = {“red,” “blue,” “green,” …}. QQ does not presuppose that all aspects of qualia can be simultaneously measured and reported^6^.

Now consider a situation where you momentarily see many color patches (Figure 2A). Suppose you are attending to the right most red patch. This kind of “sensory input” and “attention” constitute a “state,” separate from “observables.” While each color quale can be indeterminate, under a particular “state,” the expected value of a particular quale (modeled as an observable) is given. Formally, states are like functions that return the expected value for a given quale, when a particular observable is measured.

### 3.2 Dynamics of qualia observables and states: updates through instruments

In quantum theory, there are three mathematically equivalent ways to consider the dynamics of observables and states (Sakurai and Napolitano, 2020) (see Table 1 for a summary). QQ considers both observables and states to change over time. This interpretation is called an “interaction” picture.

**Table 1:** Conceptual summary of quantum terminologies (columns: observables, states, averaged measurement outcomes) and how they are used in (rows) quantum theory, quantum cognition, and QQ (the Quantum-like Qualia hypothesis).

	Observables	States	Averaged measurement outcomes
Quantum Theory	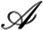	Ψ	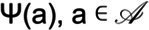
Quantum Cognition	Response options (fixed)	Mental states (dynamic)	Responses
Quantum-like Qualia	Qualia (dynamic)	Sensory inputs, attention (dynamic)	Reportable aspects of qualia

Each cell entry explains a representative usage of each concept.

In most quantum cognition studies, observables are possible response options, which are fixed, while (mental) states change dynamically. This idea of fixed-observables and dynamic-states is called the “Schrödinger” picture. In QQ, we consider sensory inputs and attention as “states.” It is not difficult to imagine how these “states” can change measurement outcomes.

In some fields of physics (e.g., particle physics), states are considered to be fixed, while observables change. This dynamic is called the “Heisenberg” picture. In QQ, it is natural to consider changes of qualia observables as a consequence of changes in the brain through perceptual learning, sensory adaptation, and so on (Song et al., 2017). In this case, even if sensory inputs and attention are fixed, qualia can change.

In this paper, we predominantly consider sensory inputs and internal attention as major foundational elements of states, but other mental elements, such as memories and expectations, can also constitute states. Thus, in this interaction picture, QQ explicitly considers how qualia (observables) interact with states (sensory inputs and attention). Without a state, we cannot consider a particular measurement outcome of any qualia observable.

Finally, to formalize how qualia observables interact with other mental processes, we introduce the concept of a “measurement instrument” (*cf.* the arrows in Figure 2; Davies and Lewis, 1970). In modern measurement theory, any measurement of the system is described by a mathematical structure called a (measurement) instrument, which offers a generalization of a conditional probability. In standard quantum physics, measurements are considered all-or-nothing. As the theory of quantum measurement matured, researchers arrived at the concept of instruments as the most general form of measurement. The formalism of instruments offers a bridge from nonlinear wave collapse (which is the result of a measurement in standard quantum theory) to the unitary dynamics of an isolated system and ‘unsharp’ or weak measurements. We propose that this generalized formalism to characterize the effects of measurements would be particularly useful when considering the interaction between qualia and attention. Attention may not determine qualia in an all-or-nothing way, but rather in an unsharp or weak way.

Instruments are utilized in modern quantum measurement theory and have started being applied in the field of quantum cognition (Khrennikov, 2015; Ozawa and Khrennikov, 2021). Instruments can describe how qualia observables and states of sensory inputs and attention dynamically develop upon measurements.

While the above descriptions are sufficient to understand the foundations of the QQ hypothesis, we now expand the conceptual framework and provide associated technical details.

### 3.3 What counts as a system?

We define qualia observables as all possible intrinsic properties of a system. But what is meant by the term “system”? We consider a system minimally as “that which is experiencing the qualia in question.” It would correspond to “the complex” in Integrated Information Theory (Albantakis et al., 2022). Over time, a system itself can change (then observables would change accordingly). Yet the system should still need to be identified as a coherent entity or phenomenon. A system has an associated set of qualia observables, which can be measured from the outer environment.

### 3.4 A state as an interface between qualia and the world

The interrelationship between the system and the environment external to it is represented by the state of the system. In a sense, a state can be considered as an interface. This idea may sound strange at first, but actually it is equally applicable across classical and quantum theory (Ojima, 2005; Saigo et al., 2019; Saigo, 2021). For example, the temperature of water in a cup as an observable needs to be determined in the context (= “state”) of where and how the measurement instruments are placed.^7^

In QQ, such a context would involve at least sensory inputs and attention. In a particular state, call it “φ,” the expected value of reporting a particular quale, P(Q = q|φ) can be established. For example, in a state φ = “one is sitting at the sunset with the mind wandering,” P(Q = “seeing the color of red” | φ) can be established. Or, in a state φ = “sensory input to a participant is a weak grating stimulus with masking under a particular attentional instruction,” we may obtain P(Q = “faint” | φ) = 0.7, when we assume Q as observables with outcomes of {highly visible, less visible, faint, not visible}. Note that in this framework, there is no point in talking about considering a single-trial quale as in [Q = “faint”] without considering the state. We can consider only an ensemble of measurement outcomes given a particular state.

The notion of an interface between system and environment is an important idea, as discussed in many theories of consciousness. Just to name a few, “interface” in interface theory of consciousness (Hoffman et al., 2015, 2023; Prakash et al., 2020; Prentner, 2021), “background conditions” in the Integrated Information Theory of consciousness (Albantakis et al., 2022), “Markov blanket” in the free energy principle (Kirchhoff et al., 2018), and “mediation” in philosophy (Taguchi, 2019).

Inspired by the mathematical structure of quantum theory, QQ aspires to establish principled associations among observables, states, and their interactions, not at the level of an individual event (or the qualia property at each moment) but at the level of collections of similar events. In fact, for every individual event, the set of all qualia properties would be unique and never identical to the other sets, especially when space and time are considered. Thus, QQ proposes that qualia should not be considered at the level that assumes definiteness of qualia properties for each event. Rather, QQ proposes to consider qualia at the level of ensembles where some “similar” qualia properties are grouped together (as in the above categorical set of observables). How to construe “similar” is an important question, which the authors have discussed elsewhere, using concepts from category theory (Tsuchiya et al., 2016, 2022, 2023). In category theory, it is quite explicit what one considers as similar is a choice of mathematicians or scientists, not automatically or uniquely ‘given’ by the world (Cheng, 2022). In most theoretical and experimental contexts, qualia are similar as long as they are considered similar in some way by the observing individual, as in the everyday usage of “similar.”

In summary, “state” is an interface that assigns an “average” value to each observable, noting that measurement of a single event may not be possible.

### 3.5 Instrument formalism for dynamics of qualia and states

Let us now consider the dynamics of qualia. For simplicity, in relation to a discrete time step, denote qualia, sensory input, and attention at time t as Q(t), S(t), and A(t). Their interdependency is illustrated by the arrows in Figure 2. The dynamical update rules are expressed as

Q(t+1)=f(Q(t),S(t),A(t)) and

A(t+1)=g(Q(t),S(t),A(t))

This simple formulation is a primitive form of an instrument. Currently, we do not have enough data to constrain the form of the functions f and g. However the equations generally formalize how changes of sensory inputs^8^ affect both what we experience and how we attend. They also capture how attending to uncertain aspects of qualia (e.g., a spatial boundary) can change qualia. For specific and empirical applications of instruments in quantum cognition, see Ozawa and Khrennikov (2021).

### 3.6 A common mathematical and philosophical structure between quantum phenomena and qualia

QQ proposes an application of some aspects of the mathematical structure from quantum theory (e.g., separation of observables, states and averaged measured outcomes, and instruments). In parallel with the mathematical structure, we surmise that there is a common philosophical stance covering both quantum phenomena and qualia. Through such a philosophical connection, QQ naturally situates some of the perplexing psychological findings in qualia and attention as detailed below.

#### 3.6.1 Noncommutativity, complementarity, uncertainty relations in quantum theory, quantum cognition, and QQ

One of the foundational ideas behind quantum theory is “complementarity.” In the context of qualia, two qualia are complementary when they cannot be experienced simultaneously, as we consider in more detail below (Bruza et al., 2023).^9^ Complementarity is a philosophical concept that one of the founders of quantum theory, Niels Bohr, introduced in physics, indirectly inspired by one of the founders of modern experimental psychology, William James, through Edgar Rubin (Holton, 1988).

The idea of complementarity can be mathematically expressed via the concept of noncommutativity (Streater, 2007; Atmanspacher and Filk, 2018). Noncommutativity implies sensitivity to the order of an operation. In general, the effect of processing A then B may not be the same as B then A. Noncommutativity is the default for many processes, from cooking to chemical reactions.^10^ In the brain, this could correspond to the effect of processing A leaving some trace, in terms of synaptic plasticity or neuronal activity, which impacts on processing B. If this is the case, processes A and B are expected to be noncommutative and likewise for the corresponding qualia.

If observables A and B are noncommutative, measuring A after B typically yields a different outcome to B after A. It is generally accepted that many aspects of human cognition are noncommutative. Even in arithmetic, subtraction and division are noncommutative. While multiplication is commutative for numbers, it is not for matrices. Note that matrix operations are fundamental to quantum theory (Busemeyer and Bruza, 2024). Noncommutative observables can be used to formalize important features of qualia, such as the aforementioned indeterminacy. Starting with the well established noncommutative formalization of quantum theory as a guiding framework, it should be possible to appropriately extend this formalism for QQ. Then, as we explain later, it should be possible to empirically demonstrate its necessity.

Regarding qualia, in general, when we consider “processes,” whereby the order of the processes matters. In an example drawn from masking, presenting target T briefly before mask M at a particular interval can make T completely invisible. But swapping the order into M then T, both of them can become highly visible. This is an example of noncommutativity. Quantitative and coherent explanations of order effects, fallacies in decision making, conceptual combination, evidence accumulation, over/under distribution effects in memory and other cognitive phenomena is one of the hallmarks of the quantum cognition framework (Busemeyer and Bruza, 2024; Busemeyer and Wang, 2017; Pothos and Busemeyer, 2022). Complementarity as noncommutativity is experimentally demonstrated as uncertainty relations (Atmanspacher and Filk, 2018).

Complementarity, noncommutativity and uncertainty relations are the basis of quantum theory, from which the field of quantum cognition arose. Quantum cognition started from explaining enigmatic phenomena in decision making (Aerts et al., 2018; Mistry et al., 2018; Basieva et al., 2019; Busemeyer et al., 2019; Broekaert et al., 2020), concept combination (Bruza et al., 2015; Wang et al., 2021; Aerts and Arguëlles, 2022), and judgment (Wang and Busemeyer, 2013; White et al., 2020; Ozawa and Khrennikov, 2021). It has recently expanded into modeling for language (Surov et al., 2021), emotion (Khrennikov, 2021; Huang et al., 2022), music (beim Graben and Blutner, 2019), and social judgments (Tesař, 2020). It is beginning to be applied to solve real-world problems (Arguëlles, 2018; Song et al., 2022; Wojciechowski et al., 2022) and it has been influencing the design of artificial intelligence and robots that aim to interact with the world (Ho and Hoorn, 2022).

To the extent that cognition is continuous with perception (Barsalou, 2010), quantum cognition is a relevant framework to consider quality of perceptual consciousness, or qualia. Indeed, certain applications of quantum cognition to perceptual judgments are already emerging (Conte et al., 2009; Atmanspacher and Filk, 2010; Asano et al., 2014; Yearsley et al., 2022; Bruza et al., 2023; Epping et al., 2023) as we will discuss below.

#### 3.6.2 A common philosophical structure between quantum phenomena and qualia

On the philosophical side, both quantum phenomena and qualia arise from “interactions.” In the above, we introduced “a state as an interface,” which is an idea almost equivalent to the philosophical concept of “mediation” (Taguchi, 2019). Quantum phenomena arise from interactions between quantum objects, such as photons, and measurement devices (Plotnitsky, 2021).

Notably, Niels Bohr stated that the “reality” responsible for quantum phenomena is indeterminate and beyond representation (Plotnitsky, 2021). By “reality,” we mean a definite single event before any measurement. Such a concept is not problematic in the classical view, which assumes that anything can exist before measurement and it is in principle not affected by measurement. In quantum theory, a property of an observable is not defined without a state and there is no meaning to a single measurement outcome. In this sense, we adopt a view analogous to Bohr’s that “reality” is “indeterminate” and “beyond representation” before any measurement.

Likewise, QQ proposes that the reality of qualia defies concrete representation in a similar way, such as points in a high dimensional space in classical models. Note that classical models can consider a distribution of points rather than a single point. However, this still assumes the existence of “reality” of qualia before measurement. Moreover, measurement is assumed to introduce noise so that a probability distribution is needed to model it. In this view, the underlying uncertainty is epistemic due to the limitation of our measurement technique or lack of knowledge. However, QQ proposes that measurement outcomes statistically arise from interrelationship between qualia observables and states of sensory inputs and attention. In other words, the underlying uncertainty of qualia is ontic due to the nature of the very “being-ness” of qualia phenomena. If qualia are ontologically uncertain, we would be unable to establish what property each qualia observable corresponds to, for at least some states at a single event, even if we had all relevant information available.^11^ For such qualia, the act of measurement does not reveal pre-existing properties of qualia observables. Rather the measured property emerges as part of the interrelationship between qualia observables and a state where a measurement takes place.

In classical philosophy literature, representationalism states that the phenomenal character of experience is reducible to representational content (Block, 1998). These views typically conceive of a definitive single event, regardless of a state, which is reduced to a cognitive representation. By contrast, anti-representational views of consciousness propose that such a definitive representation does not exist (Koenderink, 2010; Gibson, 2014; Varela et al., 2017; Schlicht and Starzak, 2021). While the precise reasoning behind the latter views is not the same, the QQ hypothesis shares the same conclusion.

The point of quantum theory, as argued by Bohr, is to abandon the assumption that “reality” must be definitive and to argue that, due to indeterminacy, the underlying “reality” cannot be represented in a classical way. Instead, quantum theory offers a suitable predictive and explanatory framework.

The analogy with qualia is that, due to their indeterminacy, some qualia cannot be “represented” as points in the dimensional space, as is usually assumed. Specifically, QQ points out that at least some qualia are indeterminate when they are in an unattended state. In many cases, when attention is directed to a particular qualia observable, measurement outcomes about the attended property would become more determinate. This corresponds to an intentional, content-bearing phenomenal object with an associated cognitive representation as proposed by the orthodox cognitive science. However, in an unattended state, these qualia observables have properties, which do not have well established values or qualities. Classical representationalism does not consider such a possibility. Further, as we elaborate later, QQ predicts that the measurement outcomes are not only statistical but they additionally violate some statistical laws that must be satisfied if qualia properties are always determinate.

### 3.7 Interim summary: what is the Quantum-like Qualia hypothesis?

In summary, QQ hypothesizes the following. First, observables correspond to all possible aspects of experience that a system can have, including experiences from all sensory modalities, as well as thoughts, concepts, memories and feelings, that is, anything, as long as it is part of an experience (i.e., qualia in the broad sense). States are a particular arrangement of the system. When the system is in a given state, averaged measurement outcomes from qualia observables can be lawfully specified. States represent sensory inputs and any internal condition of the system, including how the system attends to or accesses observables. Second, averaged measurement outcomes are results of interactions between observables and states and they can be reported outside the system. Third, observables and states change dynamically and interact with each other, as formalized by the instrument theory. From mathematical and philosophical perspectives, qualia have an analogical correspondence with quantum phenomena. Table 1 summarizes these basic concepts and how they are used in quantum theory, quantum cognition, and QQ.

## 4 What are the benefits of QQ and how can we test QQ predictions?

As explained above, QQ accords with fundamental intuitions about qualia, such as their indeterminacy, dynamics, and interaction with internal processes. Furthermore, QQ offers some important insights concerning our empirical knowledge about qualia and provides novel perspectives about the nature of qualia. Here we provide some details of three lines of investigation comprising order effects, violation of the Bell inequality, and relationships between qualia and attention, thereby showcasing how to empirically test various predictions from QQ.

### 4.1 Order effects in similarity judgments among color qualia

The QQ hypothesis is empirically testable in surprisingly simple ways. One way is to ask if the order of questions or stimuli matters for the resulting reports. Epping et al. (2023) presented a pair of color patches to participants, then asked if the reported similarities are symmetric with respect to the order of color patch presentation.

Since seminal work by Rosch (1975) and Tversky (1977), perceptual similarity judgments about colors, faces, and objects have been repeatedly shown to be asymmetric (Polk et al., 2002; Roberson et al., 2007; Hodgetts and Hahn, 2012; Best and Goldstone, 2019). These studies challenge standard points-in-space type models, requiring arguably *ad hoc* modifications (Krumhansl, 1978; Ashby and Perrin, 1988; Nosofsky, 1991).

The extremely high citation rate of Tversky’s paper attests to the fact that researchers are aware of this asymmetry. Yet, it is not common to empirically take asymmetries into account in similarity studies, as this doubles the numbers of trials. Even when different orders are included, researchers often remove them by symmetrizing the originally asymmetric similarity matrix, so that they can use popular, existing analytic algorithms, such as multidimensional scaling.

**Figure 3:**
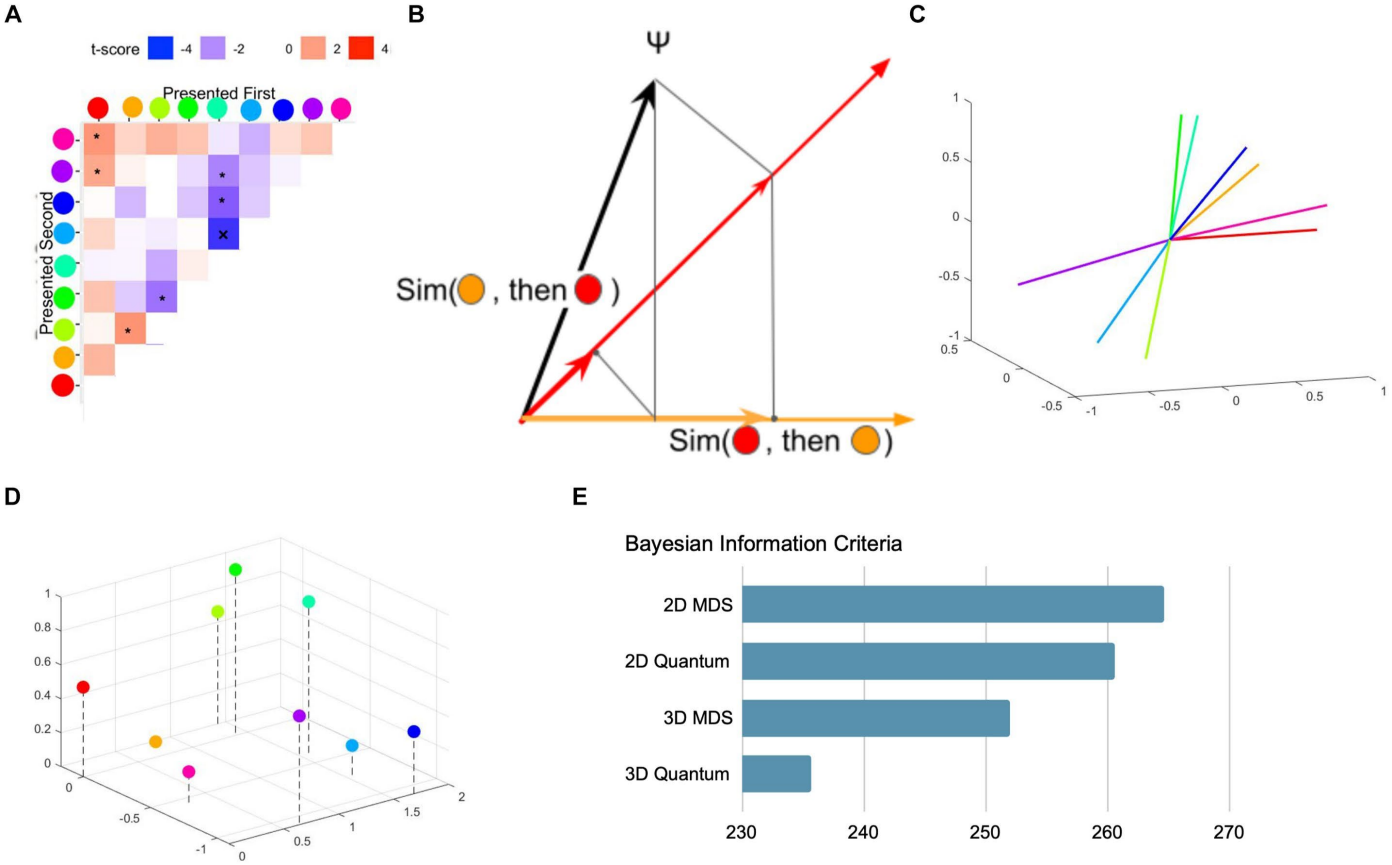
Quantum model of color similarity. **(A)** Empirical asymmetry matrix. The raw similarity matrix is subtracted from its transpose to reveal the degree of asymmetry in similarity judgments. Taken from Epping et al. (2023). **(B)** How quantum operations (projections) give rise to perceived similarity (Pothos et al., 2013; Yearsley et al., 2022; Epping et al., 2023). Assume an initial (mental) state as a unit vector Ψ (the black line). Color qualia observables {red and orange} are represented as two “subspaces” in a space (the red and orange axes). The vector is projected onto a subspace representing the color that is first experienced. From there, it is further projected onto the subspace corresponding to the second color. The resulting length of the final projection can be related to the perceived similarity between the two colors. Importantly, the resulting length can depend on the order with which the colors are experienced. **(C)** The best fit quantum similarity model for the data in **(A)** (Epping et al., 2023). In the quantum model, each of 9 color qualia observables is modeled as a subspace in 3D space. Experienced similarity between the two subspaces is related to the square value of the cosine angle between them (e.g., the red and the pink subspaces have a narrow angle, but the red and the green subspaces have a near 90 deg. angle). **(D)** Traditional 3D MDS representation of 9 colors based on their pairwise similarity. **(E)** Bayesian information criteria (BIC) for best fit 2D and 3D MDS and quantum models. Note that MDS models needed additional free parameters to account for asymmetries in similarity judgments (Nosofsky, 1991), resulting in more complex models. The 3D quantum model offered the best fit to the empirical data.

While an isolated instance of asymmetry [e.g., “Is China similar to North Korea” vs. “Is North Korea similar to China,” (Tversky, 1977)] can be explained in many possible models, a collection of perceptual reports for many stimuli, such as color patches, and a particular pattern of asymmetries across many stimuli represent a more substantial challenge (Figure 3A). Epping et al.’s quantum models, which consider a state as a density matrix (this is a generalization of the idea that a state can be a vector), and similarity as arising from sequential projections (Figure 3B), offered a better fit to the empirical data (Figure 3C), compared to points-in-space models of qualia (Figures 3D,E), with flexibility to accommodate asymmetry when mapping distance between points to similarity.

As noted previously, most similarity experiments tend to ignore the effect of order of presentation, using a simultaneous presentation paradigm, or paradigms that allow longer and uncontrolled inspection of the items. This is understandable due to the increased cost of experiments that manipulate order, because the number of the trials increases quadratically with the number of items to examine. Distributing pairs of items across many participants in online samples may solve this issue (Kawakita et al., 2023).

### 4.2 Violation of the Bell inequality in the domain of qualia

Quantum theory was developed in the 1920s by Bohr, Heisenberg, Shroedinger, Born and others. This theory challenged the predominant realist view of nature. In 1935, Einstein, Podolsky and Rosen (Einstein et al., 1935) (EPR) challenged this view, claiming that quantum theory is incomplete. In 1962, Bell discovered one fundamental inequality (Bell, 1964) must be satisfied assuming EPR’s view is correct. Subsequently, the violation of the Bell inequality was empirically demonstrated (Freedman and Clauser, 1972; Aspect et al., 1982). The Nobel Prize for Physics in 2022 was awarded for the demonstration of violations of the Bell inequality.

Since the initial EPR experiments, there has been debate about loopholes in the experiments that were being conducted. Over the years these loopholes have been successively closed. Nowadays, it is generally accepted that the EPR experiments do empirically verify that microscopic particles can violate the Bell inequalities and are therefore entangled. What this implies about the underlying nature of these particles has been debated (Zeilinger, 2010). In parallel, a classical realist view has been questioned in relation to cognitive phenomena when these violate the Bell inequalities (Bruza et al., 2023).

Bell’s inequality can be represented as follows:

$S=E\left(\mathrm{a,}\;b\right)-E\left(\mathrm{a,}\;b^{’}\right)+E\left(a^{’},\;b\right)+E\left(a^{’},\;b^{’}\right),$

where a and a’ are two measurement settings for system A, b and b’ for B, and E(:) is the expected value of the corresponding measurements. These expected values have to be measured in separate experimental conditions. In classical systems, |S| < =2, unless there are direct influences or signaling, between measurements of system A and system B. Contextuality-by-Default (CbD) is a generalization of the Bell inequalities. CbD allows a determination of contextuality in the presence of direct influences [For its application, see Basieva et al. (2019) and Cervantes and Dzhafarov (2019)]. The Bell inequality can be violated by quantum phenomena. A generally accepted explanation for the violation is that the properties of the phenomena do not have definite values at all times, that is, they are indeterminate.

For the QQ hypothesis, demonstrating that qualia violate the Bell inequality will play a similarly fundamental role. If these types of inequalities are violated, qualia can be assumed to be quantum-like (which implies additional properties, such as noncommutativity). There are many ways to psychophysically test the Bell inequalities (Basieva et al., 2019; Cervantes and Dzhafarov, 2019; Bruza et al., 2023).

#### 4.2.1 Establishing violations of the temporal Bell inequality in multistable perception

Multistable perception (Maier et al., 2012; Brascamp et al., 2018) can be used to demonstrate violations of a type of Bell inequality. Atmanspacher and Filk (2010) focused on the number of reversals between three time points of an ambiguous figure. They proposed empirical tests involving the temporal version of the Bell inequality (Yearsley and Pothos, 2014). Specifically, Atmanspacher and Filk’s proposal was to measure perceptual switches between times t1, t2, and t3, where t1 < t2 < t3, selecting two time points per condition and for all three possible combinations. The probability of the perceptual state being different at time i vs. time j is denoted by pij. If qualia are determinate at all time (as hypothesized Figure 5 and Table 1 of Atmanspacher and Filk, 2010), then it has to be the case that p12 + p23 ≥ p13. If violations of this inequality are found under some conditions, it gives reason to believe that the qualia are generally indeterminate, which is fundamental to the QQ hypothesis. (Note that qualia can be in a determinate state under some conditions under the QQ. Indeterminacy includes determinacy as a special case).

On the other hand, if qualia are generally determinate and can never be indeterminate, p12 + p23 ≥ p13 have to always apply. Without doubt, there will be many instances of qualia which indeed behave in such a classical way (as we noted above, the classical probability theory is a special case of the quantum probability theory). What is of interest is whether we can identify cases of qualia for which p12 + p23 ≥ p13 is violated. When this happens, then we can conclude that the qualia should be considered quantum-like in general (even if they might be classical-like, in many cases).^12^ The research effort for identifying such violations is still in its infancy, but there are already some promising results (Waddup et al., 2023) that showed violations of the temporal Bell inequality within a decision paradigm.

A closely related phenomenon concerns quantum Zeno effects (Atmanspacher et al., 2004; Yearsley and Pothos, 2016). Quantum Zeno effects are the surprising prediction that, everything else being equal, an increased frequency of measurements can slow down change in the relevant state. Yearsley and Pothos (2016) demonstrated the Zeno effect at the cognitive level (i.e., the switch of opinion about someone to be judged from guilty to not guilty over the accumulated evidence). If “measurements” do not affect qualia, any kind of gradual changes in qualia should not be affected by measurements. While multistable percepts change spontaneously, other types of qualia changes, such as morph-induced categorical perception and gradual change blindness, can be used to test if the effects of measurement can be precisely predicted from the quantum formulation of the Zeno effects (Atmanspacher et al., 2004; Yearsley and Pothos, 2016).

#### 4.2.2 Establishing violations of Bell inequality in multiple qualia about an object

**Figure 4:**
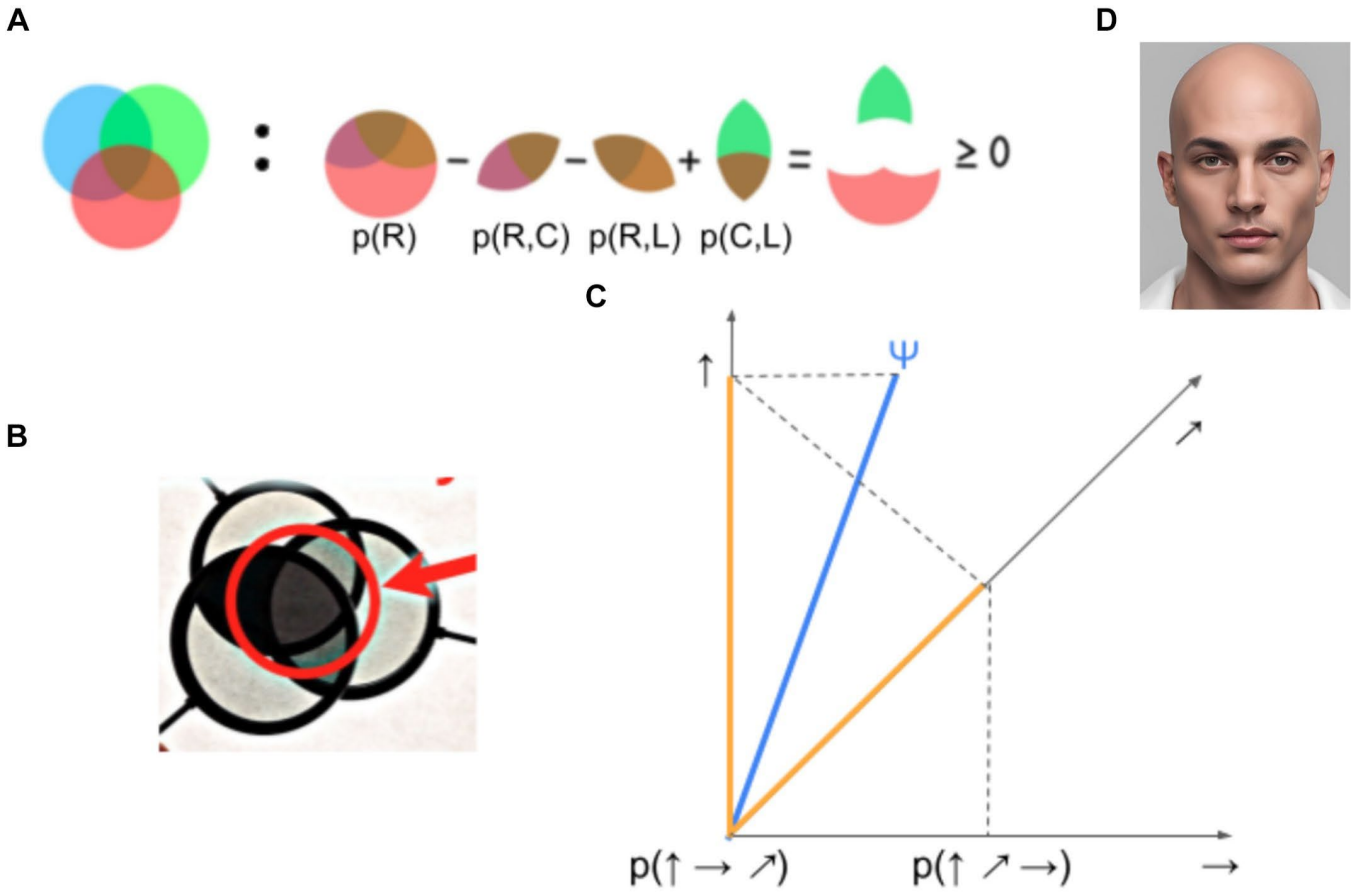
Classical probability predictions and their violations in perceptual and quantum phenomena. **(A)** Venn diagram of Boole’s idea of possible experience. **(B)** Intuitive physical demonstration of the violation of the Venn diagram constraints using polarizers. See https://www.youtube.com/watch?v=zcqZHYo7ONs. The main idea is this: prepare 3 polarizers. By arranging two of them, you can completely block any light through them. That is, the probability of passing photons across two polarizers can be set to 0. Then, insert a third polarizer between the two. Depending on the angle of the third, the three filters can pass more photons, and thus the output beam would be brighter at the intersection of the three polarizers. **(C)** An explanation of **(B)** with a quantum projection scheme. Assume the state can be influenced by measurement. After we project the initial state Ψ to the ↑ axis, further projection to the → gives 0 length, which corresponds to a perfect block of photons. However, if we project to the ↗ axis, after the ↑ one, then third projection to the → gives a non-zero length, explaining why more photons pass through three filters than just the two original ones. **(D)** An artificial face (generated by AI Canva), similar to the one used in Bruza et al. (2023), where the relationship in **(A)** does not hold for three aspects of the face (dominance, trustworthiness, and intelligence). Consequently, there is reason to believe that some of these facial traits were indeterminate prior to judgment.

Another way to test the Bell inequality is to set up a task with at least three qualia observables, measuring two observables at a time, but against three different states. If qualia can be modeled classically and if measurements do not change qualia, then we expect the logical constraints, as exemplified by a Venn diagram (Figure 4A) to be satisfied by the set of probabilities. A simple diagrammatic analysis reveals various inequalities, described by George Boole as “conditions of possible experience” (Pitowsky, 1994). Pitowsky convincingly argues that quantum phenomena violate Boole’s “conditions of possible experience” as these are predicated on an assumption of realism. As quantum phenomena do not always have definite properties at all times, like marbles being pulled from an urn, they can violate probabilistic relationships expressed in these inequalities.

Figure 4A demonstrates probability relationships among the three averaged measurement outcomes about three qualia observables, Color = {red, purple, orange, …}, Position = {up, down, center, left, right}, and Shape = {circle, octagon, hexagon,…}. Let us say, you are briefly presented with an object and you experience it with associated (narrow-sense) qualia. In classical theory, these qualia should stay the same regardless of which of two observables you report. Let Prob(C = ‘red’) = p(R), Prob(S = ‘circle’) = p(C), and Prob(P = ‘left’) = p(L) represent the probability that the averaged measurement outcomes of your qualia observables of the object is red, circular, and on the left, respectively. Then, we obtain that p(R)-p(R,C)-p(R,L) + p(C,L) has to be always non-negative. This is easily confirmed from a Venn diagram (Figure 4A).

Now, imagine the object was “masked” to reduce its visibility or two such objects are simultaneously tested. The three properties can be randomly changed from trial to trial. In such a situation, your answers are likely to become probabilistic, that is, Prob(C = ‘red’), Prob(S = ‘circle’), Prob(P = ‘left’) are all smaller than 1. But, answers will still have to satisfy various probabilistic constraints. For example, p(R)-p(R, C)-p(R, L) + p(C, L) has to be greater than or equal to 0, if these qualia properties follow the common sense assumptions regarding the objects being observed. Boole termed such probabilistic constraints “conditions of possible experience.” It is worth noting that classical intuitions regarding the averaged measurement outcomes are so entrenched, it is hard to imagine how things could be otherwise. Violations of such Venn diagram constraints can physically arise and are even easy to demonstrate in a classroom using just 3 polarizers (Figures 4B,C).^13^ This is an excellent demonstration to become familiar with the interesting reality of quantum phenomena, directly observable at the macro level.

Bruza and colleagues (Bruza et al., 2023) examined this constraint for qualia of a face. They considered three qualia observables. Whether faces appear trustworthy = {yes, no}, dominant = {yes, no}, and intelligent = {yes, no} (Figure 4D). It turned out that the Boole’s “possibility of experience” can be violated (i.e.,g p(A)-p(A,B)-p(B,C) + p(C,A) < 0), implying that the simple classic probabilistic picture in Figure 4A is inappropriate.^14^

Several extensions to the above task are possible. For example, it is plausible that the degree of violation of the Bell inequality may depend on the characteristics of the qualia. If this were the case, performing the same face experiment but with reduced visibility might induce greater violations of the Bell inequality. Visual psychophysics offer a multitude of techniques to reduce visibility of an object (Kim and Blake, 2005; Stein and Peelen, 2021). As mentioned in the opening section, one of the fundamental visibility manipulations is masking. It is interesting to note that masking among three objects (Dember and Purcell, 1967; Breitmeyer et al., 1981) has been reported to be quite complex and might reveal a promising alternative demonstration of Bell inequality violations.

One might argue that properties of faces, such as trustworthiness, dominance, and intelligence are not directly experienced qualia, but rather they are cognitively inferred constructs or concepts (Kemmerer, 2015; McClelland and Bayne, 2016). It would be a fruitful future experiment to examine if similar conclusions can be obtained when using more perceptual aspects of qualia of an object, such as color, orientation, size, location, and so on.

To sum up, one explanation for a violation of a Bell inequality is that the underlying phenomena do not have well-defined properties that exist prior to observation and distributed in a certain manner (Pitowsky, 1994). Consequently, when the inequality is violated, there is reason to believe that the phenomena are indeterminate prior to measurement. While superficially simple, definitive tests of such inequalities are subject to several checks and assumptions (Blasiak et al., 2021), and this makes it hard to definitely establish the inference from violations to indeterminacy.

While the fundamental ideas are fairly simple, almost no research on qualia has adopted a task design, where three qualia observables are measured under three states. This is understandable given that it would be difficult to motivate such a task or interpret the results, in the absence of a quantum-like theoretical framework. We believe there is a huge opportunity to test novel ideas about consciousness with the QQ formulation involving three or more observables.

### 4.3 Dual-task interference and non-interference between qualia in terms of incompatible and compatible observables

The relationship between consciousness and attention is one of the most debated topics in psychology, neuroscience and philosophy (Iwasaki, 1993; Hardcastle, 1997; Lamme, 2003; Dehaene et al., 2006; Block, 2007; Koch and Tsuchiya, 2007; Mole, 2008; van Boxtel et al., 2010a; Tallon-Baudry, 2011; Bor and Seth, 2012; Cohen et al., 2012; Pitts et al., 2018; Bronfman et al., 2019; Maier and Tsuchiya, 2021). QQ is mostly consistent with the known empirical findings. Moreover, QQ makes further testable predictions which are critical to empirical research in this area.

**Figure 5:**
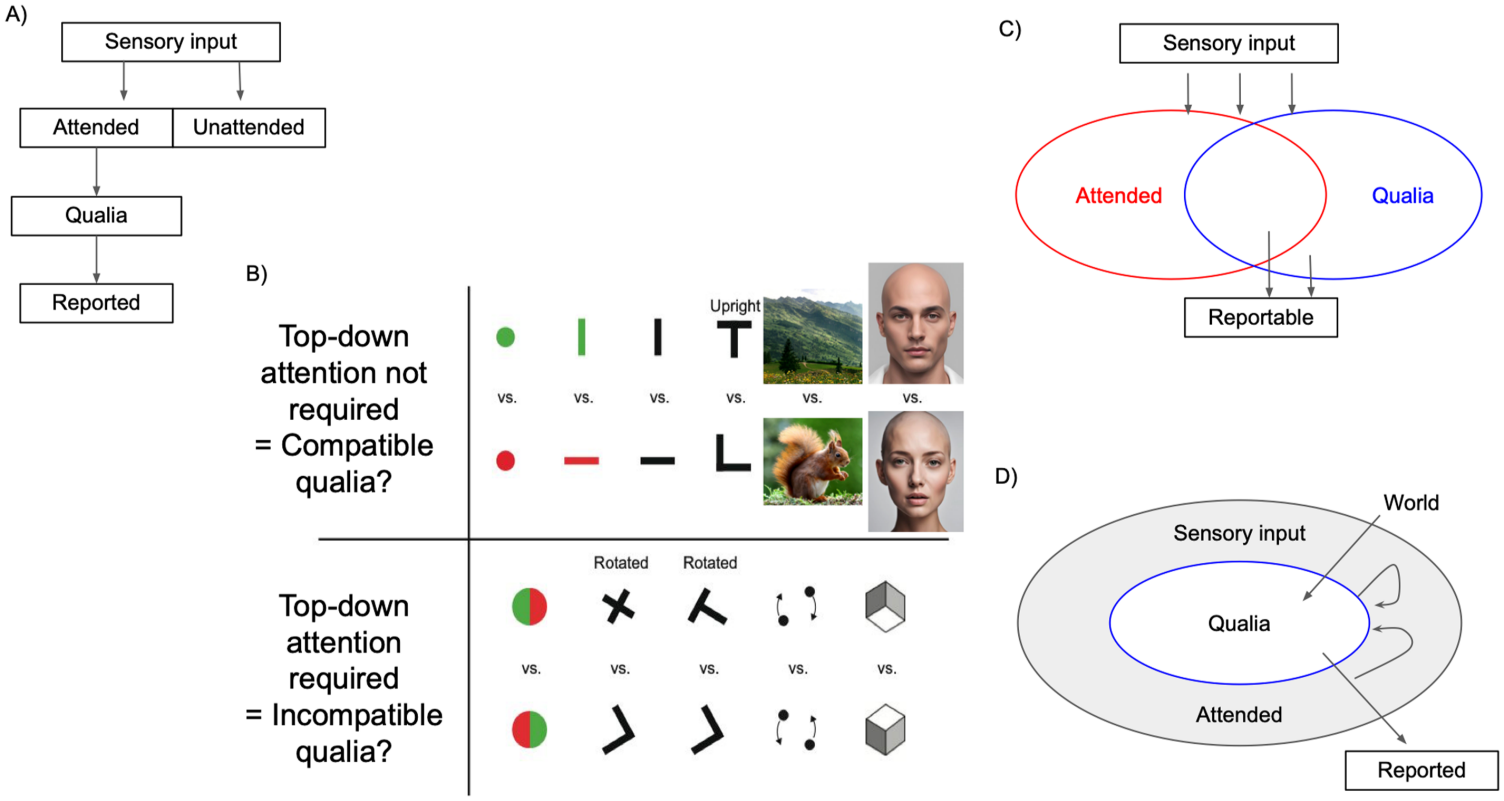
QQ is compatible with the empirical findings about the relationship between attention and qualia. **(A)** Traditional feedforward models of sensory input, attention, qualia, and reports. **(B)** Top row: a list of peripheral perceptual discriminations that can be conducted simultaneously with difficult letter discrimination tasks at the fixation. For example, conscious experience of genders presented at the periphery does not differ with or without performing a difficult central letter task (Matthews et al., 2018). Bottom row: a list of tasks that cannot be performed concurrently with the letter task. One novel interpretation of such results is using the notion of incompatibility. Incompatibility is the inability to jointly establish the values of two or more observables. Modified from Tsuchiya and Koch (2015) using faces generated by AI Canva and pictures generated by Pexels (both are free). **(C)** A static view of consciousness and attention that is consistent with dissociations between qualia and attention (Maier and Tsuchiya, 2021). **(D)** Quantum qualia hypothesis (reproduced from Figure 2).

Traditionally, sensory inputs are considered to be filtered by attention first (Figure 5A), implying that attention is necessary for consciousness. Information selected with attention is experienced as qualia and subsequently reported in a feedforward manner. Only some aspects of sensory input are attended, which ostensibly give rise to particular qualia. Behavioral reports reflect the experienced qualia. In this model, typically, attention is considered as a single limited resource and any task consumes some amount of attention.

This view goes against empirical findings concerning reports of sensory inputs outside of attention. Among many empirical findings, a particularly intriguing one is a pattern of tasks that consume almost all attention and those that do not consume any attention, as shown in Figure 5B. These properties of task combinations have been documented over the years within the “dual task” research program (Braun and Sagi, 1990; Braun and Julesz, 1998; Reddy et al., 2004; Fei-Fei et al., 2005; Pastukhov et al., 2009; Matthews et al., 2018; Bronfman et al., 2019). For example, conscious experience of genders presented at the periphery do not differ with or without performing a difficult central letter task. Meanwhile, the experience of red/green bisected disks becomes totally unclear under a dual-task with the same central task (Reddy et al., 2004, 2006). Notably, this is even the case when the disk and the face are superposed transparently at the same location (Matthews et al., 2018). One possible explanation of this pattern is the existence of attention-free specialized modules in the cortex, possibly due to biological significance or extended training (VanRullen et al., 2004).

There are many alternatives to the traditional view of attention and consciousness. One view considers consciousness and attention to operate independently (Figure 5C; Lamme, 2004; Koch and Tsuchiya, 2007). In this scheme, unattended conscious and attended unconscious processes are both possible. Attention and consciousness do not proceed in a feedforward manner. While this view is consistent with empirical findings, it does not explain how consciousness and attention interact dynamically.

The QQ hypothesis (Figures 2, 5D) explicitly considers how qualia can be affected by attention through the formalism of instruments. This does not mean that all qualia are equally affected by attention, as demonstrated by the dual task. In fact, QQ provides two novel explanations about why a given pair of tasks may not interfere with one another.

One explanation has to do with the existence of “commutative” qualia. While any process is generally noncommutative (see 3.6.1), in quantum theory, some observables, called “centers,” are always commutative with any other observables. Centers do not show any order effects. Such observables include mass. It is plausible that some types of qualia (e.g., extreme pain, bright light, loud sound) may also behave like centers and be commutative with other types of qualia. These would also be predicted to be less affected by states of measurement including attention. This is an empirical question for future research, which can be addressed by testing the presence of order effects in similarity experiments, for example.

Another explanation relates to the idea of “incompatibility.” In quantum theory, when the properties of two or more observables cannot not be generally established together, these observables are called “incompatible.” According to QQ, pairs of qualia observables that cannot be simultaneously established are deemed “incompatible.”

From the QQ perspective, it is important to point out that, in many dual tasks, a letter discrimination task is used as the primary difficult fixation task (Tsuchiya and Koch, 2015; Matthews et al., 2018). Thus, the conclusions from these studies may be revealing “incompatibility” between qualia observables of letters and others. In other words, some qualia observables, such as face gender (Matthews et al., 2018) and the presence of animals in a natural scene (Li et al., 2002; Figure 5B top row), may just be “compatible” with a letter qualia observable. These qualia observables may be “incompatible” with others. If the attentional interference happens only at the task level, we should not expect systematic patterns in interference and order effects. However, if interference is a result of the incompatibility between specific qualia combinations, then interference would result in specific order effects with a quantitative explanation based on a quantum-like model (Epping et al., 2023).

Reconsidering the patterns of attentional limits in terms of incompatibilities between observables might allow novel insights into the qualia-attention research. With traditional psychological theories, we consider attention as a fixed resource (Joseph et al., 1997), which can amplify aspects of qualia, it is hard to explain why in some visual illusions stronger attention leads to poorer visibility of the target (Schölvinck and Rees, 2009; van Boxtel et al., 2010b). Further, it is also hard to understand why distracting participants sometimes leads to better psychological performance in various paradigms (Koch and Tsuchiya, 2007; Tsuchiya and Koch, 2015). Attention can change the neuronal circuitry momentarily (Harris and Thiele, 2011; Gilbert and Li, 2013), thus it might be possible to understand such effects as a change, for a pair of observables, from incompatible into compatible. This change can be formalized as an instrument where attention as a state affects qualia observables. This explanation offers a coherent explanation of these seemingly odd relationships between qualia and attention.

Unlike the limited resource model, QQ predicts an existence of pairs of “compatible” qualia observables, even though each one consumes a significant amount of a presumed attentional “resource.” QQ also predicts pairs of “incompatible” qualia observables, which cannot be simultaneously established, even if each does not consume much attentional resource. Discoveries of such pairs of qualia observables would further support QQ.

## 5 Conclusion

We proposed a Quantum-like Qualia (QQ) hypothesis based on a quantum theoretical framework (e.g., noncommutative observables, states, and instruments; Figure 2; Table 1). QQ proposes qualia as observables, not the “things” or results of “cognitive processes” as traditionally assumed. QQ explains intuitive and known properties of qualia, such as their inherent indeterminacy, dynamics, and interaction with attention. Predictions from QQ can be empirically tested with demonstrations of asymmetry in perceptual similarity judgments, violations of the Bell inequality, and apparent incompatibilities between particular qualia. Among these, particularly powerful are demonstrations of Bell inequality violations. In order to test them, we minimally need to measure three observables, two at a time across three different states (Figure 4). Such experiments have been rarely conducted systematically, due to the lack of theoretical background and motivation. Additionally, there are subtle loopholes that need to be considered, before compelling empirical evidence is provided that substantiates our claim that qualia are indeterminate (Emary, 2017; Atmanspacher and Filk, 2019; Basieva et al., 2019). In physics, it took more than twenty years from the theoretical proposal by Bell through to the initial experiment by Clauser and then to the compelling demonstration by Aspect (Section 4.2.1). Will a similar pathway await the Quantum-like Qualia hypothesis in the future? Only time will tell. With increasing evidence that QQ provides a coherent explanation on the mathematical structure of qualia, QQ may well emerge as a promising mathematical and philosophical framework to link qualia and the brain.

## Footnotes

^1^Temporally extended and varying qualia can be represented as either a dynamically moving single point in high-dimensional space or a single point of a very high-dimensional space, where different time points are represented as different dimensions.

^2^One promising venue is dynamical models of consciousness and qualia (Fekete and Edelman, 2011; Esteban et al., 2018; Moyal et al., 2020). However, so far, such models do not address the issue of how measurements and observations affect qualia, one of the central points of our paper.

^3^For more recent mathematically elaborated models, see Hoffman et al. (2023), , Kleiner (2024), , Kleiner and Ludwig (2024) and references therein.

^4^Some studies in quantum cognition are highly relevant to our proposal (Filk, 2009; Khrennikov, 2015, 2021; Atmanspacher and Müller-Herold, 2016). Our Quantum-like Qualia (QQ) hypothesis is quite orthogonal to the Quantum Brain hypothesis, which considers quantum mechanical processes in the brain (Hameroff and Penrose, 2014) and the role of consciousness in quantum collapse (Chalmers and McQueen, 2021) (see also Smolin, 2022). QQ is completely consistent with the possibility that all physical events happening in the brain are purely classical. Our core idea is to utilize the mathematical formalism of quantum theory, as outlined below. For these and other related issues see Atmanspacher (2017).

^5^This is different from the quantum question (QQ) equality by Wang et al. (2014).

^6^Note this statement is about measurement and reports on qualia. We assume that qualia exist before measurements in the same way quantum particles exist before measurement.

^7^Consider all possible temperatures of water as observables. The temperature of water is a complex physical concept, which depends not only on the average kinetic energy of water molecules but also on the measuring probe device’s temperature, surface areas, and many other factors. We treat all of these factors that relate to measurement as “states.” In the case of measuring water temperature, depending on how invasive the measurement probe is (with a probe from either a very cold or very hot environment), the measured outcome of the temperature of water can change.

^8^While some theories consider a possible role of conscious agents on the control of S(t + 1) through motor control and intention, we consider that they are better left out from the formalism of this update rule of instrument for qualia. Consider the sensory input while you are looking at an ever-changing shape and colors of a burning fireplace. Also, in an experimental situation, experimenters can change sensory input S(t + 1) to a participant in any way they want.

^9^Note that we are not saying that all qualia are complementary to each other. At least some combinations of qualia are likely to be complementary and cannot be experienced at the same time. Indeed, at each moment, we are experiencing multiple qualia at the same time. This is consistent with our introduction of a concept of “broad-sense” qualia. A broad-sense quale is composed of qualia in narrow sense in a unified way.

^10^Note that non-commutativity includes commutativity as a special case. This is similar to the statement that quantum probability theory includes classical probability theory as a special case.

^11^As “ontologically” indeterminate qualia, we consider several cases where measurements of qualia have non-ignorable impacts (periphery, similarity judgements, attention related experiments). In Section 4, we provided empirical experiments to address this issue. In classical physics objects exist independent of measurement. Similarly, classical qualia models tend to assume existence of qualia independent of measurement. For example, in encountering an unfamiliar painting, classical models tend to assume that you have some preference even if you do not articulate it or even if it is uncertain. Our QQ is more explicit about this. Some qualia are affected by measurement and measurement instrument theory (in the future) should specify how a particular type of measurement should affect qualia in what way. This also means that QQ also anticipates some qualia are not affected by measurements as well (say, the color of apple in front of you).

^12^It is worth repeating here that even if we were to find violations of temporal Bell inequality, it does not mean that brains that support qualia are operating in non-classical mechanisms. Instead, it would exclude mathematical structures for qualia that are purely based on classical notions (e.g., determinacy). Rather more broader mathematical structures, such as quantum-like, need to be considered.

^13^https://www.youtube.com/watch?v=zcqZHYo7ONs

^14^Note that this does not mean that the quantum-like explanation is unique and the only way to explain this result. Rather, quantum theory is able to bring together a body of insights and mechanisms, in a coherent, axiomatic framework.

## References

[ref1] Aerts D. Arguëlles J. A. (2022). Human perception as a phenomenon of quantization. Entropy 24:1207. doi: 10.3390/e24091207, PMID: 36141092 PMC9497542

[ref2] Aerts D. Arguëlles J. A. Beltran L. Geriente S. Sassoli de Bianchi M. Sozzo S.. (2018). Spin and wind directions I: identifying entanglement in nature and cognition. Found. Sci. 23, 323–335. doi: 10.1007/s10699-017-9528-9, PMID: 29805293 PMC5960007

[ref3] Albantakis L. Barbosa L. Findlay G. Grasso M. Haun A. M. Marshall W.. (2022). Integrated information theory (IIT) 4.0: Formulating the properties of phenomenal existence in physical terms. PLOS Computational Biology. 19, e1011465. doi: 10.1371/journal.pcbi.1011465 PMC10581496 37847724

[ref4] Arguëlles J. A. (2018). The heart of an image: quantum superposition and entanglement in visual perception. Found. Sci. 23, 757–778. doi: 10.1007/s10699-018-9547-1

[ref5] Asano M. Hashimoto T. Khrennikov A. Ohya M. Tanaka Y. (2014). Violation of contextual generalization of the Leggett–Garg inequality for recognition of ambiguous figures. Phys. Scr. T163:014006. doi: 10.1088/0031-8949/2014/T163/014006

[ref6] Ashby F. G. Perrin N. A. (1988). Toward a unified theory of similarity and recognition. Psychol. Rev. 95, 124–150. doi: 10.1037/0033-295X.95.1.124

[ref7] Aspect A. Dalibard J. Roger G. (1982). Experimental test of Bell’s inequalities using time-varying analyzers. Phys. Rev. Lett. 49, 1804–1807. doi: 10.1103/PhysRevLett.49.1804

[ref8] Atmanspacher H. (2017). “Quantum approaches to brain and mind: an overview with representative examples” in The Blackwell companion to consciousness. eds. Schneider S. Velmans M.. 1st ed (Hoboken, NJ: Wiley), 298–313.

[ref9] Atmanspacher H. Filk T. (2010). A proposed test of temporal nonlocality in bistable perception. J. Math. Psychol. 54, 314–321. doi: 10.1016/j.jmp.2009.12.001

[ref10] Atmanspacher H. Filk T. (2018). Non-commutativity and its implications in physics and beyond. Orpheus’ Glance. Selected Papers on Process Psychology: The Fontarèches Meetings, 2002–2017, 45–60.

[ref11] Atmanspacher H. Filk T. (2019). Contextuality revisited: signaling may differ from communicating. In Barros J. A. de Montemayor C. (Eds.), Quanta and mind (Vol. 414, pp. 117–127). New York: Springer International Publishing.

[ref12] Atmanspacher H. Filk T. Römer H. (2004). Quantum Zeno features of bistable perception. Biol. Cybern. 90, 33–40. doi: 10.1007/s00422-003-0436-4, PMID: 14762722

[ref13] Atmanspacher H. Müller-Herold U. (2016). From chemistry to consciousness. New York: Springer International Publishing.

[ref14] Bachmann T. (2000). Microgenetic approach to the conscious mind, vol. 25. Amsterdam: John Benjamins Publishing.

[ref15] Balduzzi D. Tononi G. (2009). Qualia: the geometry of integrated information. PLoS Comput. Biol. 5:e1000462. doi: 10.1371/journal.pcbi.1000462, PMID: 19680424 PMC2713405

[ref16] Barsalou L. W. (2010). Grounded cognition: past, present, and future: topics in cognitive science. Top. Cogn. Sci. 2, 716–724. doi: 10.1111/j.1756-8765.2010.01115.x, PMID: 25164052

[ref17] Basieva I. Cervantes V. H. Dzhafarov E. N. Khrennikov A. (2019). True contextuality beats direct influences in human decision making. J. Exp. Psychol. Gen. 148, 1925–1937. doi: 10.1037/xge0000585, PMID: 31021152

[ref18] beim Graben P. Blutner R. (2019). Quantum approaches to music cognition. J. Math. Psychol. 91, 38–50. doi: 10.1016/j.jmp.2019.03.002

[ref19] Bell J. S. (1964). On the einstein podolsky rosen paradox. Physics Physique Fizika 1, 195–200. doi: 10.1103/PhysicsPhysiqueFizika.1.195

[ref20] Best R. M. Goldstone R. L. (2019). Bias to (and away from) the extreme: comparing two models of categorical perception effects. J. Exp. Psychol. Learn. Mem. Cogn. 45, 1166–1176. doi: 10.1037/xlm0000609, PMID: 30024260

[ref21] Blasiak P. Pothos E. M. Yearsley J. M. Gallus C. Borsuk E. (2021). Violations of locality and free choice are equivalent resources in Bell experiments. Proc. Natl. Acad. Sci. 118:e2020569118. doi: 10.1073/pnas.2020569118, PMID: 33888585 PMC8092485

[ref22] Block N. (1998). Is experiencing just representing? Philos. Phenomenol. Res. 58, 663–670. doi: 10.2307/2653766

[ref23] Block N. (2007). Consciousness, accessibility, and the mesh between psychology and neuroscience. Behav. Brain Sci. 30, 481–499. doi: 10.1017/S0140525X07002786 18366828

[ref24] Bor D. Seth A. K. (2012). Consciousness and the prefrontal parietal network: insights from attention, working memory, and chunking. Front. Psychol. 3:63. doi: 10.3389/fpsyg.2012.00063, PMID: 22416238 PMC3298966

[ref25] Borg I. Groenen P. J. F. (2005). Modern multidimensional scaling: Theory and applications. 2nd Edn. Berlin: Springer.

[ref26] Brascamp J. Sterzer P. Blake R. Knapen T. (2018). Multistable perception and the role of the Frontoparietal cortex in perceptual inference. Annu. Rev. Psychol. 69, 77–103. doi: 10.1146/annurev-psych-010417-085944, PMID: 28854000

[ref27] Braun J. Julesz B. (1998). Withdrawing attention at little or no cost: detection and discrimination tasks. Percept. Psychophys. 60, 1–23. doi: 10.3758/BF03211915 9503909

[ref28] Braun J. Sagi D. (1990). Vision outside the focus of attention. Percept. Psychophys. 48, 45–58. doi: 10.3758/BF03205010 2377439

[ref29] Breitmeyer B. G. Ogmen H. (2007). Visual masking. Scholarpedia 2:3330. doi: 10.4249/scholarpedia.3330

[ref30] Breitmeyer B. G. Rudd M. Dunn K. (1981). Metacontrast investigations of sustained-transient channel inhibitory interactions. J. Exp. Psychol. Hum. Percept. Perform. 7, 770–779. doi: 10.1037/0096-1523.7.4.770, PMID: 6457091

[ref31] Broekaert J. B. Busemeyer J. R. Pothos E. M. (2020). The disjunction effect in two-stage simulated gambles. An experimental study and comparison of a heuristic logistic, Markov and quantum-like model. Cogn. Psychol. 117:101262. doi: 10.1016/j.cogpsych.2019.101262, PMID: 31865226

[ref32] Bronfman Z. Z. Jacobson H. Usher M. (2019). Impoverished or rich consciousness outside attentional focus: recent data tip the balance for overflow. Mind Lang. 34, 423–444. doi: 10.1111/mila.12217

[ref33] Bruza P. D. Fell L. Hoyte P. Dehdashti S. Obeid A. Gibson A.. (2023). Contextuality and context-sensitivity in probabilistic models of cognition. Cogn. Psychol. 140:101529. doi: 10.1016/j.cogpsych.2022.101529, PMID: 36476378

[ref34] Bruza P. D. Kitto K. Ramm B. J. Sitbon L. (2015). A probabilistic framework for analysing the compositionality of conceptual combinations. J. Math. Psychol. 67, 26–38. doi: 10.1016/j.jmp.2015.06.002

[ref35] Bujack R. Teti E. Miller J. Caffrey E. Turton T. L. (2022). The non-Riemannian nature of perceptual color space. Proc. Natl. Acad. Sci. 119:e2119753119. doi: 10.1073/pnas.2119753119, PMID: 35486695 PMC9170152

[ref36] Busemeyer J. R. Bruza P. D. (2024). Quantum models of cognition and decision. Cambridge: Cambridge University Press.

[ref37] Busemeyer J. R. Kvam P. D. Pleskac T. J. (2019). Markov versus quantum dynamic models of belief change during evidence monitoring. Sci. Rep. 9:18025. doi: 10.1038/s41598-019-54383-9, PMID: 31792262 PMC6889126

[ref38] Busemeyer J. R. Wang Z. (2017). Is there a problem with quantum models of psychological measurements? PLoS One 12:e0187733. doi: 10.1371/journal.pone.0187733, PMID: 29117246 PMC5678685

[ref39] Carrasco M. Barbot A. (2019). Spatial attention alters visual appearance. Curr. Opin. Psychol. 29, 56–64. doi: 10.1016/j.copsyc.2018.10.010, PMID: 30572280 PMC7661009

[ref40] Casarotto S. Comanducci A. Rosanova M. Sarasso S. Fecchio M. Napolitani M.. (2016). Stratification of unresponsive patients by an independently validated index of brain complexity. Ann. Neurol. 80, 718–729. doi: 10.1002/ana.24779, PMID: 27717082 PMC5132045

[ref41] Cervantes V. H. Dzhafarov E. N. (2019). True contextuality in a psychophysical experiment. J. Math. Psychol. 91, 119–127. doi: 10.1016/j.jmp.2019.04.006

[ref42] Chalmers D. J. McQueen K. J. (2021). Consciousness and the collapse of the wave function. ArXiv. doi: 10.48550/arXiv.2105.02314

[ref43] Cheng E. (2022). The joy of abstraction an exploration of math, category theory, and life. Cambridge: Cambridge University Press.

[ref44] Churchland P. (2005). Chimerical colors: some phenomenological predictions from cognitive neuroscience. Philos. Psychol. 18, 527–560. doi: 10.1080/09515080500264115

[ref45] Clark A. (2000). A theory of sentience. Oxford: Oxford University Press.

[ref46] Cohen M. A. Cavanagh P. Chun M. M. Nakayama K. (2012). The attentional requirements of consciousness. Trends Cogn. Sci. 16, 411–417. doi: 10.1016/j.tics.2012.06.013 22795561

[ref47] Conte E. Khrennikov A. Y. Todarello O. Federici A. Mendolicchio L. Zbilut J. P. (2009). Mental states follow quantum mechanics during perception and cognition of ambiguous figures. Open Syst. Inform. Dyn. 16, 85–100. doi: 10.1142/S1230161209000074

[ref48] Cowen A. S. Fang X. Sauter D. Keltner D. (2020). What music makes us feel: at least 13 dimensions organize subjective experiences associated with music across different cultures. Proc. Natl. Acad. Sci. 117, 1924–1934. doi: 10.1073/pnas.1910704117, PMID: 31907316 PMC6995018

[ref49] Cowen A. S. Keltner D. (2017). Self-report captures 27 distinct categories of emotion bridged by continuous gradients. Proc. Natl. Acad. Sci. 114, E7900–E7909. doi: 10.1073/pnas.1702247114, PMID: 28874542 PMC5617253

[ref50] Davies E. B. Lewis J. T. (1970). An operational approach to quantum probability. Commun. Math. Phys. 17, 239–260. doi: 10.1007/BF01647093

[ref51] Dehaene S. Changeux J. P. Naccache L. Sackur J. Sergent C. (2006). Conscious, preconscious, and subliminal processing: a testable taxonomy. TICS 10, 204–211. doi: 10.1016/j.tics.2006.03.007 16603406

[ref52] Dember W. N. Purcell D. G. (1967). Recovery of masked visual targets by inhibition of the masking stimulus. Science 157, 1335–1336. doi: 10.1126/science.157.3794.1335, PMID: 6039004

[ref53] Einstein A. Podolsky B. Rosen N. (1935). Can quantum-mechanical description of physical reality be considered complete? Phys. Rev. 47, 777–780. doi: 10.1103/PhysRev.47.777

[ref54] Emary C. (2017). Ambiguous measurements, signalling and violations of Leggett-Garg inequalities. Phys. Rev. A 96:042102. doi: 10.1103/PhysRevA.96.042102

[ref55] Epping G. P. Fisher E. L. Zeleznikow-Johnston A. M. Pothos E. M. Tsuchiya N. (2023). A quantum geometric framework for modeling color similarity judgments. Cogn. Sci. 47:e13231. doi: 10.1111/cogs.13231, PMID: 36655940

[ref56] Esteban F. J. Galadí J. A. Langa J. A. Portillo J. R. Soler-Toscano F. (2018). Informational structures: a dynamical system approach for integrated information. PLoS Comput. Biol. 14:e1006154. doi: 10.1371/journal.pcbi.1006154 30212467 PMC6161919

[ref57] Exner S. (1868). Über die zu einer Gesichtswahrnehmung nöthige Zeit. Wiener Sitzungsber Math-Naturwissensch Cl Kaiser Akad Wissensch 58, 601–632.

[ref58] Fei-Fei L. VanRullen R. Koch C. Perona P. (2005). Why does natural scene categorization require little attention? Exploring attentional requirements for natural and synthetic stimuli. Vis. Cogn. 12, 893–924. doi: 10.1080/13506280444000571

[ref59] Fekete T. Edelman S. (2011). Towards a computational theory of experience. Conscious. Cogn. 20, 807–827. doi: 10.1016/j.concog.2011.02.010 21388834

[ref60] Filk T. (2009). Quantum physics and consciousness: the quest for a common, modified conceptual foundation. Mind Matter 7, 59–79.

[ref61] Filk T. (2013). Temporal non-locality. Found. Phys. 43, 533–547. doi: 10.1007/s10701-012-9671-7

[ref62] Freedman S. J. Clauser J. F. (1972). Experimental test of local hidden-variable theories. Phys. Rev. Lett. 28, 938–941. doi: 10.1103/PhysRevLett.28.938

[ref63] Gärdenfors P. (2000). “Conceptual spaces: The geometry of thought” in A Bradford Book (Cambridge, MA: MIT Press).

[ref64] Gibson J. J. (2014). The ecological approach to visual perception. Classic Edn. London: Psychology press.

[ref65] Gilbert C. D. Li W. (2013). Top-down influences on visual processing. Nat. Rev. Neurosci. 14, 350–363. doi: 10.1038/nrn3476, PMID: 23595013 PMC3864796

[ref66] Graham D. J. Friedenberg J. D. Rockmore D. N. Field D. J. (2010). Mapping the similarity space of paintings: image statistics and visual perception. Vis. Cogn. 18, 559–573. doi: 10.1080/13506280902934454

[ref67] Hameroff S. Penrose R. (2014). Consciousness in the universe: a review of the ‘Orch OR’theory. Phys Life Rev 11, 39–78. doi: 10.1016/j.plrev.2013.08.002 24070914

[ref68] Hardcastle V. G. (1997). Attention versus consciousness: a distinction with a difference. Cogn. Stud. Bull. Jpn. Cogn. Sci. Soc. 4, 56–66.

[ref69] Harris K. D. Thiele A. (2011). Cortical state and attention. Nat. Rev. Neurosci. 12, 509–523. doi: 10.1038/nrn3084, PMID: 21829219 PMC3324821

[ref70] Haven E. Khrennikov A. (2013). Quantum Social Science. 1st Edn. Cambridge: Cambridge University Press.

[ref71] Hebart M. N. Zheng C. Y. Pereira F. Baker C. I. (2020). Revealing the multidimensional mental representations of natural objects underlying human similarity judgements. Nat. Hum. Behav. 4, 1173–1185. doi: 10.1038/s41562-020-00951-3, PMID: 33046861 PMC7666026

[ref72] Ho J. K. W. Hoorn J. F. (2022). Quantum affective processes for multidimensional decision-making. Sci. Rep. 12:20468. doi: 10.1038/s41598-022-22855-0, PMID: 36443304 PMC9705568

[ref73] Hodgetts C. J. Hahn U. (2012). Similarity-based asymmetries in perceptual matching. Acta Psychol. 139, 291–299. doi: 10.1016/j.actpsy.2011.12.003 22305350

[ref74] Hoffman D. D. Prakash C. Prentner R. (2023). Fusions of consciousness. Entropy 25:129. doi: 10.3390/e25010129, PMID: 36673270 PMC9858210

[ref75] Hoffman D. D. Singh M. Prakash C. (2015). The Interface theory of perception. Psychon. Bull. Rev. 22, 1480–1506. doi: 10.3758/s13423-015-0890-8 26384988

[ref76] Holton G. (1988). “The Roots of Complementarity.” In Quantum Mechanics, Routledge. 253–93.

[ref77] Huang J. A. Zhang Q. Busemeyer J. R. Breithaupt F. (2022). A quantum walk model for emotion transmission in serial reproduction of narratives. Proceedings of the Annual Meeting of the Cognitive Science Society. 44. Available at: https://escholarship.org/uc/item/2tj1w6hw

[ref78] Indow T. (1988). Multidimensional studies of Munsell color solid. Psychol. Rev. 95, 456–470. doi: 10.1037/0033-295X.95.4.456, PMID: 3057527

[ref79] Iwasaki S. (1993). Spatial attention and two modes of visual consciousness. Cognition 49, 211–233. doi: 10.1016/0010-0277(93)90005-G, PMID: 8131376

[ref80] Joseph J. S. Chun M. M. Nakayama K. (1997). Attentional requirements in a “preattentive” feature search task. Nature 387, 805–807. doi: 10.1038/42940, PMID: 9194560

[ref81] Kahneman D. (2003). Experiences of collaborative research. Am. Psychol. 58, 723–730. doi: 10.1037/0003-066X.58.9.723 14584989

[ref82] Kanai R. Tsuchiya N. (2012). Qualia. Curr. Biol. 22, R392–R396. doi: 10.1016/j.cub.2012.03.033 22625852

[ref83] Kawakita G. Zeleznikow-Johnston A. Takeda K. Tsuchiya N. Oizumi M. (2023). Is my “red” your “red”?: unsupervised alignment of qualia structures via optimal transport. PsyArXiv. doi: 10.31234/osf.io/h3pqm

[ref84] Kemmerer D. (2015). Are we ever aware of concepts? A critical question for the global neuronal workspace, integrated information, and attended intermediate-level representation theories of consciousness. Neurosci. Consciousness 2015:6. doi: 10.1093/nc/niv006, PMID: 30135741 PMC6089087

[ref85] Khrennikov A. Y. (2010). Ubiquitous Quantum Structure. Berlin: Springer Berlin Heidelberg.

[ref86] Khrennikov A. (2015). Quantum-like model of unconscious–conscious dynamics. Front. Psychol. 6:997. doi: 10.3389/fpsyg.2015.00997, PMID: 26283979 PMC4522519

[ref87] Khrennikov A. (2021). Quantum-like model for unconscious–conscious interaction and emotional coloring of perceptions and other conscious experiences. Biosystems 208:104471. doi: 10.1016/j.biosystems.2021.104471, PMID: 34237350

[ref88] Kim C. Y. Blake R. (2005). Psychophysical magic: rendering the visible ‘invisible’. TICS 9, 381–388. doi: 10.1016/j.tics.2005.06.012 16006172

[ref89] Kirchhoff M. Parr T. Palacios E. Friston K. Kiverstein J. (2018). The Markov blankets of life: autonomy, active inference and the free energy principle. J. R. Soc. Interface 15:20170792. doi: 10.1098/rsif.2017.0792, PMID: 29343629 PMC5805980

[ref90] Kleiner J. (2024). Towards a structural turn in consciousness science. Conscious. Cogn. 119:103653. doi: 10.1016/j.concog.2024.103653, PMID: 38422757

[ref91] Kleiner J. Ludwig T. (2024). What is a mathematical structure of conscious experience? Synthese 203:89. doi: 10.1007/s11229-024-04503-4

[ref92] Klincewicz M. (2011). “Quality space model of temporal perception” in Multidisciplinary aspects of time and time perception. eds. Vatakis A. Esposito A. Giagkou M. Cummins F. Papadelis G., vol. 6789 (Berlin: Springer Berlin Heidelberg), 230–245.

[ref93] Koch C. Massimini M. Boly M. Tononi G. (2016). Neural correlates of consciousness: Progress and problems. Nat. Rev. Neuro. 17, 307–321. doi: 10.1038/nrn.2016.22, PMID: 27094080

[ref94] Koch C. Tsuchiya N. (2007). Attention and consciousness: two distinct brain processes. Trends Cogn. Sci. 11, 16–22. doi: 10.1016/j.tics.2006.10.012 17129748

[ref95] Koenderink J. J. (2010). “Vision and information” in Perception beyond inference: The information content of visual processes (Cambridge, MA: MIT Press), 27–57.

[ref96] Kostic D. (2012). The vagueness constraint and the quality space for pain. Philos. Psychol. 25, 929–939. doi: 10.1080/09515089.2011.633696

[ref97] Krumhansl C. L. (1978). Concerning the Applicability of Geometric Models to Similarity Data: The Interrelationship between Similarity and Spatial Density. 85, 445–163.

[ref98] Lamme V. A. (2003). Why visual attention and awareness are different. Trends Cogn. Sci. 7, 12–18. doi: 10.1016/S1364-6613(02)00013-X, PMID: 12517353

[ref99] Lamme V. A. (2004). Separate neural definitions of visual consciousness and visual attention; a case for phenomenal awareness. Neural Netw. 17, 861–872. doi: 10.1016/j.neunet.2004.02.005, PMID: 15288903

[ref100] Lee A. Y. (2021). Modeling Mental Qualities. Philos. Rev. 130, 263–298. doi: 10.1215/00318108-8809919

[ref101] Li F. F. VanRullen R. Koch C. Perona P. (2002). Rapid natural scene categorization in the near absence of attention. Proc. Natl. Acad. Sci. USA 99, 9596–9601. doi: 10.1073/pnas.092277599, PMID: 12077298 PMC123186

[ref9004] Lin L. (2023). The Relational Structure of Emotional Experience: A Novel Paradigm for Characterizing Differences Between Alexithymic and General Online Participants. Zenodo. doi: 10.5281/zenodo.10252326, PMID: 36004320

[ref102] Lyre H. (2022). Neurophenomenal structuralism. A philosophical agenda for a structuralist neuroscience of consciousness. Neurosci. Consciousness 2022:niac012. doi: 10.1093/nc/niac012, PMID: 36004320 PMC9396309

[ref103] Maier A. Panagiotaropoulos T. I. Tsuchiya N. Keliris G. A. (2012). Introduction to research topic—binocular rivalry: a gateway to studying consciousness. Front. Hum. Neurosci. 6:263. doi: 10.3389/fnhum.2012.00263, PMID: 23055962 PMC3457016

[ref104] Maier A. Tsuchiya N. (2021). Growing evidence for separate neural mechanisms for attention and consciousness. Atten. Percept. Psychophys. 83, 558–576. doi: 10.3758/s13414-020-02146-4, PMID: 33034851 PMC7886945

[ref105] Mashour G. A. Roelfsema P. Changeux J.-P. Dehaene S. (2020). Conscious processing and the global neuronal workspace hypothesis. Neuron 105, 776–798. doi: 10.1016/j.neuron.2020.01.026, PMID: 32135090 PMC8770991

[ref106] Matthews J. Schroder P. Kaunitz L. van Boxtel J. J. A. Tsuchiya N. (2018). Conscious access in the near absence of attention: critical extensions on the dual-task paradigm. Philos. Trans. R. Soc. Lond. Ser. B Biol. Sci. 373:352. doi: 10.1098/rstb.2017.0352, PMID: 30061465 PMC6074075

[ref107] McClelland T. Bayne T. (2016). Concepts, contents, and consciousness. Neurosci. Conscious. 2016:12. doi: 10.1093/nc/niv012, PMID: 30135743 PMC6089095

[ref108] Melloni L. Mudrik L. Pitts M. Koch C. (2021). Making the hard problem of consciousness easier. Science 372, 911–912. doi: 10.1126/science.abj3259 34045342

[ref109] Melloni L. Schwiedrzik C. M. Muller N. Rodriguez E. Singer W. (2011). Expectations change the signatures and timing of electrophysiological correlates of perceptual awareness. J. Neurosci. 31, 1386–1396. doi: 10.1523/JNEUROSCI.4570-10.2011 21273423 PMC6623627

[ref110] Mistry P. K. Pothos E. M. Vandekerckhove J. Trueblood J. S. (2018). A quantum probability account of individual differences in causal reasoning. J. Math. Psychol. 87, 76–97. doi: 10.1016/j.jmp.2018.09.003

[ref111] Mole C. (2008). Attention in the absence of consciousness? Trends Cogn. Sci. 12:44. doi: 10.1016/j.tics.2007.11.001 18178514

[ref112] Moyal R. Fekete T. Edelman S. (2020). Dynamical emergence theory (DET): a computational account of phenomenal consciousness. Mind. Mach. 30, 1–21. doi: 10.1007/s11023-020-09516-9

[ref113] Nosofsky R. M. (1991). Stimulus bias, asymmetric similarity, and classification. Cogn. Psychol. 23, 94–140. doi: 10.1016/0010-0285(91)90004-8

[ref114] Nummenmaa L. Hari R. Hietanen J. K. Glerean E. (2018). Maps of subjective feelings. Proc. Natl. Acad. Sci. 115, 9198–9203. doi: 10.1073/pnas.1807390115, PMID: 30154159 PMC6140475

[ref115] Ojima I. (2005). “Micro-Macro Duality in Quantum Physics.” in Stochastic Analysis: Classical and Quantum, WORLD SCIENTIFIC, 143–161. doi: 10.1142/9789812701541_0012

[ref116] Ozawa M. Khrennikov A. (2021). Modeling combination of question order effect, response replicability effect, and QQ-equality with quantum instruments. J. Math. Psychol. 100:102491. doi: 10.1016/j.jmp.2020.102491

[ref117] Pastukhov A. Fischer L. Braun J. (2009). Visual attention is a single, integrated resource. Vis. Res. 49, 1166–1173. doi: 10.1016/j.visres.2008.04.011 18514756

[ref118] Pitowsky I. (1994). George Boole’s ‘conditions of possible experience’ and the quantum puzzle. Br. J. Philos. Sci. 45, 95–125. doi: 10.1093/bjps/45.1.95

[ref119] Pitts M. A. Lutsyshyna L. A. Hillyard S. A. (2018). The relationship between attention and consciousness: an expanded taxonomy and implications for ‘no-report’ paradigms. Philos. Trans. Royal Society B 373:20170348. doi: 10.1098/rstb.2017.0348, PMID: 30061462 PMC6074089

[ref120] Plotnitsky A. (2021). Reality without realism: Matter, thought, and Technology in Quantum Physics. New York: Springer International Publishing.

[ref121] Polk T. A. Behensky C. Gonzalez R. Smith E. E. (2002). Rating the similarity of simple perceptual stimuli: asymmetries induced by manipulating exposure frequency. Cognition 82, B75–B88. doi: 10.1016/S0010-0277(01)00151-2, PMID: 11747865

[ref122] Pothos E. M. Busemeyer J. R. (2022). Quantum Cognition. Annu. Rev. Psychol. 73, 749–778. doi: 10.1146/annurev-psych-033020-123501 34546804

[ref123] Pothos E. M. Busemeyer J. R. Trueblood J. S. (2013). A quantum geometric model of similarity. Psychol. Rev. 120, 679–696. doi: 10.1037/a0033142 23915087

[ref124] Prakash C. Fields C. Hoffman D. D. Prentner R. Singh M. (2020). Fact, fiction, and fitness. Entropy 22:514. doi: 10.3390/e22050514, PMID: 33286286 PMC7517005

[ref125] Prentner R. (2021). Dr Goff, tear down this wall! The Interface theory of perception and the science of ConscDr Goff, tear down this wall! The Interface theory of perception and the science of Consciousnessiousness. J. Conscious. Stud. 28, 91–103. doi: 10.53765/20512201.28.9.091

[ref126] Reddy L. Reddy L. Koch C. (2006). Face identification in the near-absence of focal attention. Vis. Res. 46, 2336–2343. doi: 10.1016/j.visres.2006.01.020, PMID: 16542699

[ref127] Reddy L. Wilken P. Koch C. (2004). Face-gender discrimination is possible in the near-absence of attention. J. Vis. 4, 106–117. doi: 10.1167/4.2.4, PMID: 15005651

[ref128] Renero A. (2014). Consciousness and mental qualities for auditory sensations. J. Conscious. Stud. 21, 179–204. doi: 10.53765/20512201.21.9.179

[ref130] Roberson D. Damjanovic L. Pilling M. (2007). Categorical perception of facial expressions: evidence for a “category adjustment” model. Mem. Cogn. 35, 1814–1829. doi: 10.3758/BF03193512, PMID: 18062556

[ref131] Rosch E. (1975). Cognitive reference points. Cogn. Psychol. 7, 532–547. doi: 10.1016/0010-0285(75)90021-3

[ref132] Rosenthal D. (2015). “Quality spaces and sensory modalities” in Phenomenal qualities. eds. Coates P. Coleman S. (Oxford: Oxford University Press), 33–65.

[ref133] Saigo H. (2021). Quantum Fields as category algebras. Symmetry 13:9. doi: 10.3390/sym13091727

[ref134] Saigo H. Naruse M. Okamura K. Hori H. Ojima I. (2019). Analysis of soft robotics based on the concept of category of mobility. Complexity 2019, 1–12. doi: 10.1155/2019/1490541

[ref135] Sakurai J. Napolitano J. (2020). Modern Quantum Mechanics (3rd ed.). Cambridge: Cambridge University Press.

[ref136] Schlicht T. Starzak T. (2021). Prospects of enactivist approaches to intentionality and cognition. Synthese 198, 89–113. doi: 10.1007/s11229-019-02361-z

[ref137] Schölvinck M. L. Rees G. (2009). Attentional influences on the dynamics of motion-induced blindness. J. Vis. 9, 38–38.9. doi: 10.1167/9.1.38, PMID: 19271908 PMC2654968

[ref138] Seth A. K. Bayne T. (2022). Theories of consciousness. Nat. Rev. Neurosci. 23, 439–452. doi: 10.1038/s41583-022-00587-4 35505255

[ref139] Shepard R. N. (1962a). The analysis of proximities: multidimensional scaling with an unknown distance function. I. Psychometrika 27, 125–140. doi: 10.1007/BF02289630

[ref140] Shepard R. N. (1962b). The analysis of proximities: multidimensional scaling with an unknown distance function. II. Psychometrika 27, 219–246. doi: 10.1007/BF02289621

[ref141] Shepard R. N. Susan C. (1970). “Second-Order Isomorphism of Internal Representations: Shapes of States.” Cognitive Psychology. 1, 1–17.

[ref142] Shepard R. N. (1980). Multidimensional scaling, tree-fitting, and clustering. Science 210, 390–398. doi: 10.1126/science.210.4468.390, PMID: 17837406

[ref143] Shepard R. N. (1982). Geometrical approximations to the structure of musical pitch. Psychol. Rev. 89, 305–333. doi: 10.1037/0033-295X.89.4.305 7134331

[ref144] Shepard R. N. (1987). Toward a universal law of generalization for psychological science. Science 237, 1317–1323. doi: 10.1126/science.3629243 3629243

[ref145] Shepard R. N. Cooper L. A. (1992). Representation of colors in the blind, color-blind, and normally sighted. Psychol. Sci. 3, 97–104. doi: 10.1111/j.1467-9280.1992.tb00006.x

[ref146] Simons D. J. Rensink R. A. (2005). Change blindness: past, present, and future. Trends Cogn. Sci. 9, 16–20. doi: 10.1016/j.tics.2004.11.006, PMID: 15639436

[ref147] Smolin L. (2022). “On the place of qualia in a relational universe” in Consciousness and quantum mechanics. ed. Gao S. (Oxford: Oxford University Press), 01.

[ref148] Song C. Haun A. M. Tononi G. (2017). Plasticity in the structure of visual space. Eneuro 4:ENEURO.0080-17.2017. doi: 10.1523/ENEURO.0080-17.2017, PMID: 28660245 PMC5482114

[ref149] Song Q. Wang W. Fu W. Sun Y. Wang D. Gao Z. (2022). Research on quantum cognition in autonomous driving. Sci. Rep. 12:300. doi: 10.1038/s41598-021-04239-y, PMID: 34997146 PMC8741815

[ref150] Stein T. Peelen M. V. (2021). Dissociating conscious and unconscious influences on visual detection effects. Nat. Hum. Behav. 5, 612–624. doi: 10.1038/s41562-020-01004-5, PMID: 33398144

[ref151] Streater (2007). Lost causes in and beyond physics. Berlin: Springer Berlin Heidelberg.

[ref152] Surov I. A. Semenenko E. Platonov A. V. Bessmertny I. A. Galofaro F. Toffano Z.. (2021). Quantum semantics of text perception. Sci. Rep. 11:4193. doi: 10.1038/s41598-021-83490-9, PMID: 33603018 PMC7893056

[ref153] Taguchi S. (2019). Mediation based phenomenology. Metodo 7, 17–44. doi: 10.19079/metodo.7.2.17

[ref154] Tallon-Baudry C. (2011). On the neural mechanisms subserving consciousness and attention. Front. Psychol. 2:397. doi: 10.3389/fpsyg.2011.00397, PMID: 22291674 PMC3253412

[ref155] Tesař J. (2020). A quantum model of strategic decision-making explains the disjunction effect in the Prisoner’s dilemma game. Decision 7, 43–54. doi: 10.1037/dec0000110

[ref156] Tsuchiya N. Koch C. (2015). “The relationship between consciousness and top-down attention” in The neurology of consciousness. eds. Laureys S. Tononi G. Gosseries O.. 2nd ed (Cambridge, MA: Academic Press), 69–89.

[ref157] Tsuchiya N. Phillips S. Saigo H. (2022). Enriched category as a model of qualia structure based on similarity judgements. Conscious. Cogn. 101:103319. doi: 10.1016/j.concog.2022.103319, PMID: 35436717

[ref158] Tsuchiya N. Saigo H. (2021). A relational approach to consciousness: categories of level and contents of consciousness. Neurosci. Consciousness 2021:34. doi: 10.1093/nc/niab034, PMID: 34659799 PMC8517618

[ref159] Tsuchiya N. Saigo H. Phillips S. (2023). An adjunction hypothesis between qualia and reports. Front. Psychol. 13:1053977. doi: 10.3389/fpsyg.2022.1053977, PMID: 37077507 PMC10107370

[ref160] Tsuchiya N. Taguchi S. Saigo H. (2016). Using category theory to assess the relationship between consciousness and integrated information theory. Neurosci. Res. 107, 1–7. doi: 10.1016/j.neures.2015.12.007, PMID: 26748074

[ref161] Tversky A. (1977). Features of similarity. Psychol. Rev. 84, 327–352. doi: 10.1037/0033-295X.84.4.327

[ref162] Tye M. (2021). “Qualia” in The Stanford encyclopedia of philosophy (fall 2021). ed. Zalta E. N. (Stanford, CA: Stanford University).

[ref163] Tyler C. W. (2015). Peripheral color demo. I-Perception 6:204166951561367. doi: 10.1177/2041669515613671, PMID: 27551354 PMC4975120

[ref164] van Boxtel J. J. Tsuchiya N. Koch C. (2010a). Consciousness and attention: on sufficiency and necessity. Front. Psychol. 1:217. doi: 10.3389/fpsyg.2010.00217, PMID: 21833272 PMC3153822

[ref165] van Boxtel J. J. Tsuchiya N. Koch C. (2010b). Opposing effects of attention and consciousness on afterimages. PNAS 107, 8883–8888. doi: 10.1073/pnas.0913292107, PMID: 20424112 PMC2889341

[ref167] VanRullen R. Reddy L. Koch C. (2004). Visual search and dual tasks reveal two distinct attentional resources. J. Cogn. Neurosci. 16, 4–14. doi: 10.1162/089892904322755502, PMID: 15006031

[ref168] Varela F. Evan Thompson J. Rosch E. (2017). The embodied Mind_ cognitive science and human experience. 2nd Edn. Cambridge, MA: The MIT Press.

[ref169] Waddup O. J. Yearsley J. M. Blasiak P. Pothos E. M. (2023). Temporal Bell inequalities in cognition. Psychon. Bull. Rev. 30, 1946–1953. doi: 10.3758/s13423-023-02275-5, PMID: 37069421 PMC10716061

[ref170] Wang Z. Busemeyer J. R. (2013). A quantum question order model supported by empirical tests of an a priori and precise prediction. Top. Cogn. Sci. 5, 689–710. doi: 10.1111/tops.12040, PMID: 24027203

[ref171] Wang D. Sadrzadeh M. Abramsky S. Cervantes V. H. (2021). On the quantum-like Contextuality of ambiguous phrases (arXiv:2107.14589). arXiv. doi: 10.48550/arXiv.2107.14589

[ref172] Wang Z. Solloway T. Shiffrin R. M. Busemeyer J. R. (2014). Context effects produced by question orders reveal quantum nature of human judgments. Proc. Natl. Acad. Sci. 111, 9431–9436. doi: 10.1073/pnas.1407756111, PMID: 24979797 PMC4084470

[ref173] White L. C. Pothos E. M. Jarrett M. (2020). The cost of asking: how evaluations Bias subsequent judgments. Decision 7, 259–286. doi: 10.1037/dec0000136

[ref174] Wojciechowski B. W. Izydorczyk B. Blasiak P. Yearsley J. M. White L. C. Pothos E. M. (2022). Constructive biases in clinical judgment. Top. Cogn. Sci. 14, 508–527. doi: 10.1111/tops.12547, PMID: 34080786

[ref175] Yearsley J. M. Pothos E. M. (2014). Challenging the classical notion of time in cognition: a quantum perspective. Proc. R. Soc. B Biol. Sci. 281:20133056. doi: 10.1098/rspb.2013.3056, PMID: 24598421 PMC3953843

[ref176] Yearsley J. M. Pothos E. M. (2016). Zeno’s paradox in decision-making. Proc. R. Soc. B Biol. Sci. 283:20160291. doi: 10.1098/rspb.2016.0291 PMC4843661 27053743

[ref177] Yearsley J. M. Pothos E. M. Barque-Duran A. Trueblood J. S. Hampton J. A. (2022). Context effects in similarity judgments. J. Exp. Psychol. Gen. 151, 711–717. doi: 10.1037/xge0001097 34472962

[ref178] Young B. Keller A. Rosenthal D. (2014). Quality-space theory in olfaction. Front. Psychol. 5:1. doi: 10.3389/fpsyg.2014.00001, PMID: 24474945 PMC3893576

[ref179] Zeilinger A. (2010). Dance of the photons: From Einstein to quantum teleportation. New York: Farrar, Straus and Giroux.

[ref180] Zeleznikow-Johnston A. Aizawa Y. Yamada M. Tsuchiya N. (2023). Are color experiences the same across the visual Field? J. Cogn. Neurosci. 35, 509–542. doi: 10.1162/jocn_a_01962, PMID: 36638234

